# Aromatic Characterization of New White Wine Varieties Made from Monastrell Grapes Grown in South-Eastern Spain

**DOI:** 10.3390/molecules25173917

**Published:** 2020-08-27

**Authors:** Juan Daniel Moreno-Olivares, Maria José Giménez-Bañón, Diego Fernando Paladines-Quezada, Jose Cayetano Gómez-Martínez, Ana Cebrián-Pérez, Jose Ignacio Fernández-Fernández, Juan Antonio Bleda-Sánchez, Rocio Gil-Muñoz

**Affiliations:** Instituto Murciano de Investigación y Desarrollo Agrario y Alimentario (IMIDA), Ctra. La Alberca s/n, 30150 Murcia, Spain; juand.moreno5@carm.es (J.D.M.-O.); mariaj.gimenez8@carm.es (M.J.G.-B.); diegopaladines@hotmail.com (D.F.P.-Q.); josec.gomez2@carm.es (J.C.G.-M.); ana.cebrian@carm.es (A.C.-P.); josei.fernandez@carm.es (J.I.F.-F.); juanantonio.bleda@carm.es (J.A.B.-S.)

**Keywords:** aromatic profile, crosses, HS-SPME-GC-MS, sensorial analysis, white wines

## Abstract

The aromatic profile of a wine is one of the main characteristics appreciated by consumers. Due to climate change, vineyards need to adapt to new conditions, and one of the strategies that might be followed is to develop new white varieties from Monastrell and other cultivars by means of intervarietal crosses, since white varieties are a minority in south-eastern Spain. Such crosses have already been obtained and have been seen to provide quality white wines of high acidity and with a good aromatic composition. To confirm this, a quantitative analysis was carried out during two vintages (2018 and 2019) in order to study and compare the volatile composition of Verdejo (V) wine with the aromatic composition of several wines made from different crosses between Cabernet Sauvignon (C), Syrah (S), Tempranillo (T), and Verdejo (V) with Monastrell (M), by means of headspace SPME-GC-MS analysis. Wine volatile compounds (alcohols, volatile acids, ethyl esters, terpenes, norisoprenoids, and two other compounds belonging to a miscellaneous group) were identified and quantified using a HS-SPME-GS-MS methodology. An additional sensory analysis was carried out by a qualified tasting panel in order to characterize the different wines. The results highlighted how the crosses MT103, MC69, and MC180 showed significant differences from and better quality than the Verdejo wine. These crosses produced higher concentrations of several aromatic families analyzed, which was supported by the views of the tasting panel, thus confirming their excellent aromatic potential as cultivars for producing grapes well adapted to this area for making white wines.

## 1. Introduction

The historical tradition of winemaking in the province of Murcia (south-eastern Spain) is characterized by the main variety of red grape used, Monastrell, which represents 90% of the wine produced with the DOP Jumilla. Aromatically, this variety is characterized by an organoleptic descriptor defined as “sweet” [1], while having aromas of spicy and balsamic notes and an abundance of the odorant compounds associated with red and black fruits. By contrast, Airen, one of the main white varieties grown in this area, only represents a low percentage (<10%) of DOP Jumilla wines. This variety is characterized by its slight aroma and a medium fruity character [2]. However, one of the main varieties cultivated in Spain, which is associated with quality wines worldwide, is Verdejo. Indeed, this variety represents 5% of the white varieties grown in the whole of Spain. For example, it is the main variety behind the Denomination of Origin “Rueda” from Castilla y León (central Spain), and gives the white wine its genuineness and characteristic quality, characterized by its typical fruity aroma and flavor [3].

Climate change is a real problem with many implicit and dire consequences for many areas of the world [4]. A recent report from the Intergovernmental Panel on Climate Change (IPCC) [5] shows that, since the end of the 19th century, the mean surface temperature of the earth has risen continuously, reaching approximately 1 °C above pre-industrial levels in 2017 (mean increase of 0.2 °C per decade) due to past and current emissions derived from human activity (IPCC, 2018). This has had a noticeable effect on grape cultivars and, as a consequence, the wine made from them, due to the time elapsing between the technological maturity of grapes and their phenolic maturity. Moreover, the total acidity of the wines has tended to decrease because of the higher temperatures, resulting in unbalanced wines of less color and higher alcoholic graduation. The problems of climate change, therefore, will play an important role in the future of viticulture and oenology and new strategies need to be found which will help the industry to adapt to this new scenario.

There are several options that might attempt to solve this situation, such as selecting the plant material, patterns, and varieties best adapted to hot dry climates, or choosing the most efficient irrigation systems in the face of water scarcity. Another possible strategy could be to obtain new varieties using directed intervarietal crosses through classical genetic improvement programs, although cross-breeding is not the most common way for developing new wine grape cultivars because of European Union restrictions concerning the introduction of new varieties of vine. In this respect, a breeding program has been in progress since 2000 in Murcia (south-eastern Spain) carried out by several researchers using different approaches. For example, Bayo-Canha et al. [6] carried out a genetic analysis of grape high-quality ripening in the ‘Monastrell’ × ‘Syrah’ progeny, and Gil-Muñoz et al. [7] studied the concentration of anthocyanins in Monastrell grape hybrids and the corresponding wines. Apolinar-Valiente et al. [8] reported the composition of cell walls in grape skin from intraspecific *Vitis vinifera* hybrids and Gomez-Plaza et al. [9] studied the anthocyanin profile of intraspecific *Vitis Vinifera* hybrids (Monastrell × Cabernet Sauvignon) among others. The program uses Monastrell (mother) crossed with other varieties (used as parents), such as Tempranillo, Cabernet Sauvignon, Syrah, and Verdejo in an attempt to improve Monastrell quality wines. At the beginning, the aim was to increase the phenolic composition of these new red varieties, but, surprisingly, new white varieties emerged during the study, which were daughters of these crosses with red varieties. Therefore, the adaptation and possible marketing of these white crosses adapted to the new climatological conditions of the area (high temperatures in summer and little precipitation) were considered.

The moment of harvest is an important parameter that should be taken into account to ensure the best aromatic characteristics of a wine. For example, the degree of maturity will decide the alcohol content in wines, a parameter of great importance from a sensorial point of view since wine with high alcohol content has numerous organoleptic consequences, including a decrease in freshness and a change in the perception of the aromatic bouquet [10]. Thus, climate change-related variations in grape ripening can also modify the aromatic perception of wines, either directly, as a result of the formation of compounds characterized by over-ripe fruit notes or a reduction in vegetal, fresh, and flowery notes [11], or indirectly, through modification of the aromatic profile due to the increased alcohol content [10]. Although the best wines in many regions have traditionally been produced in the warmest years, the projected increases in average temperature and climate variability during forthcoming decades may threaten the competitive advantage some regions [12], including Murcia, which is already marked by high average temperatures. The impact of temperature on grape ripening has been studied by several authors; for example, Sadras et al. [13] tried bringing forward vintage times in order to decrease the alcoholic grade and preserve aromas, while Robison et al. [14] discussed the biochemical and chemical origins of wine volatiles and the effects of climate and viticultural practices, and Rice et al. [15] looked into the effect of harvesting time on the volatile composition of Briana and Frontenac Gris grapes. Furthermore, wines with a lower alcohol satisfy the demands of consumers, who also perceive that high alcohol levels affect a wine’s sensory perceptions, leading to unbalanced wines [16].

To sum up, although some studies have been published in recent years on the determination of polyphenolic compounds in red crosses from Monastrell in grape juice and wine [8], little information exists on aromatic composition of new white varieties in our area. For this reason, the purpose of this work was to quantitatively characterize the aromatic composition of these new varieties, in order to discover whether they meet the aromatic quality standards or even exceed those of Verdejo wines, and secondly, to ascertain whether the moment of harvest is important for their characterization and composition, and as well the adaptation of these new varieties to the new climatic scenario.

## 2. Results and Discussion

### 2.1. General Grape and Wine Parameters

Table 1 and Table 2 highlight the data at the two ripeness levels analyzed both in 2018 and 2019, and data which might have had a great impact on wine quality and aroma as well. The results obtained for the date of harvest, when the physico-chemical parameters of the musts (Brix, pH, total acidity, tartaric acid, and malic acid) were measured, and for the end of alcoholic fermentation (alcohol, pH, and total acidity), pointed to different trends for the wines made from Verdejo and the white crosses.

As can be seen in Table 1 for the musts harvested in 2018, the highest Brix values were provided by the crosses MC180, MS30, MS82, and MV67, while the MV11 cross, along with Verdejo grape, showed the lowest values, differences that would be partly related with the intrinsic characteristics of each grape variety [17], as all the cultivars were planted in the same experimental field with the same climatic conditions. As regards total acidity, MS30 and MC69 were the most acidic musts due to their high tartaric and malic acid contents, while Verdejo grape and MS33 had the lowest values, both in terms of acidity and their tartaric and malic acid contents. It is important to take into account that the accumulation of total soluble sugars and the decrease in total acidity occur faster at the beginning of veraison and more slowly as maturity approaches [18], and that the different samples may have been at different moments of ripening, since, despite being different varieties and growing in the same edaphoclimatic conditions, there was only a difference of one week between the first variety to be harvested and the last (Table 1). On the other hand, MS30, MS82, and MT103 obtained the lowest pH values, which was not unexpected because they contained the highest concentrations of tartaric acid than the other crosses and, especially, Verdejo. It should be noted that MC69 had the highest concentration of malic acid (3.77 g L^−1^), exceeding the Verdejo variety by more than one point, in spite of having a high Brix (22.30). This suggests that it might be considered a variety well adapted to the climatic conditions of south-eastern Spain due to its correct acidity and correct maturity. As regards the wine parameters analyzed, the percentage of alcohol measured in most of the wines was higher than 13.5%, while MS33 wine reached 13.76%; by contrast, MV11 and Verdejo wines reached 12.7% and 12.9%, respectively. The wine with the highest acidity was that made from MS82, although all of them were above 6.5 g/L. Finally, the pH of all the wines was below 3.5, and can be considered microbiologically suitable in our area. In short, the physicochemical parameters of grapes and wines suggest that the new varieties analyzed would be good candidates for obtaining quality wines.

Table 2 shows the data obtained in 2019. It should be pointed out that the moment of harvest took place approximately one week before that of 2018, thereby lowering the Brix in musts for the purpose of studying the influence of this factor on the volatile composition of the resulting wines. During the 2018 season (Table 1), grapes were harvested at technological maturity (around 21–22 Brix), but in 2019 (Table 2) they were harvested before reaching this stage in order to consider this factor in the aromatic profile. All the varieties were harvested with Brix values of below 20, the MS30 must having the lowest value (17.3) and MT103, MV11, and Verdejo musts the highest values but never reaching 20 Brix. It is known that ethanol production increases with the sugar content of berries (the higher the content, the greater the production of ethanol), so that the volatility of odor-active compounds in wine decreases, and fruity aromas change to alcohol-associated aromas [15]. This suggests that harvesting grapes with a lower Brix could help growers to obtain grapes with organoleptic characteristics that are more suitable for the elaboration of quality white wines.

The total acidity had high values in all the analyzed varieties, and in 2019 it was not necessary to adjust the acidity of the musts because the concentrations were significantly higher in most varieties, values ranging from 4.54 for MS33 to 7.38 for MC69, obtaining values significantly higher than that obtained for the Verdejo variety, in which tartaric acid reached a value of 5.26 g/L. In addition, as can be seen in Table 2, malic acid concentrations were greater than 2 g/L in nine of the white crosses analyzed and in Verdejo. As this compound is considered as a measure of quality, so it can be observed how the concentration of the malic acid was overcome with high differences for MC69 cross in both years (Table 1 and Table 2), possibly imparting an added value in terms of the final wine quality. The concentrations in tartaric and malic acids (the accumulation of which may have evolved to combat the effects of aging) are related with acidity and are therefore critical for wine quality [19]. In our case, the results for the musts differed as a function of the year analyzed and, as was to be expected, total acidity was lower in wines elaborated in 2018 than in 2019.

Another aspect to bear in mind is the moment of harvest and its correlation with climate change and its consequences; for example, the acidic decrease in grapes during maturation period: So many studies have been carried out on different grape varieties and have demonstrated that one of the main consequences of abiotic stress in grapes resulting from this change is an acidity decrease in grapes during the maturation period [20], so it is important to highlight the good adaptation of these crosses to the new climatic scenario. Despite the high temperatures reached during both years (close to 40 °C) and the low rainfall (around 100–140 mm between April and September), good levels of acidity were obtained, especially in 2019 (data not shown). Pre-veraison malic acid biosynthesis is fastest at 20–25 °C, whereas post-veraison malate degradation continues to accelerate up to a maximum of 50 °C [12], and so it is very important to control this parameter in order to reach the optimal concentration of malic acid. In our study, in order to be selected as a potential new white variety well adapted to the new scenario resulting from climate change, malic acid had to be higher than 2 g L^−1^ or at least be close to 2 g/L^−1^. This might be why the malic acid concentration was lower in 2018, the main difference between the two vintages being the moment of harvest. As mentioned above, malic acid degrades during the ripening process and so the more mature the grapes are, the lower the levels of malic acid. Furthermore, in some cases the lower concentrations of malic acid are also due to its lower concentrations in berries, especially in grapes grown in warmer regions. Whatever the case, any factor that affects vine growth and/or physiology directly or indirectly impacts on the fruit composition, which results in wide quality variations from one year to another [12].

As regards the analytical parameters, the wines from 2019 had, as expected, less alcohol (ranging from 10.04% for MS30 cross to 12.16% for MT103 cross). It is important to note that all the wines had a lower alcohol content in 2019, so the main objective was to obtain wines with a lower alcoholic content. For this year, in order to compare with the previous one, again, the pH of all the wines was lower than 3.5, ensuring their microbiological stability.

### 2.2. Concentration of Total Volatile Compounds

**Figure 1:**
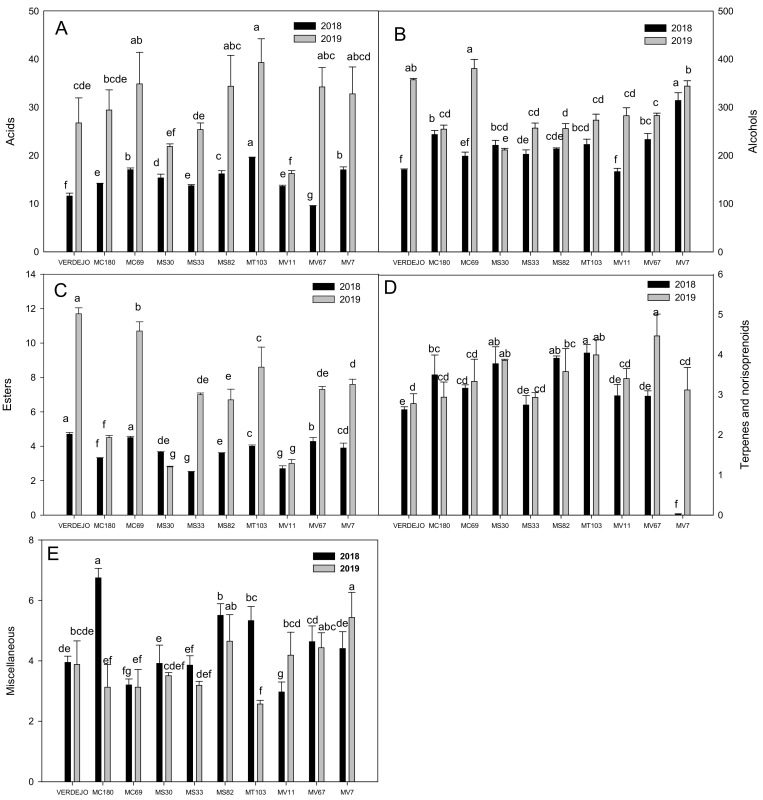
Total concentration of volatile compounds within the different aromatic families: Acids (**A**), alcohols (**B**), esters (**C**), terpenes and norisoprenoids (**D**), and miscellaneous (**E**). Comparison between the different crosses and Verdejo as control, in two different seasons (2018 in black and 2019 in grey) expressed as mg/L (ppm). Different letters are specific to each year and show significant differences (ANOVA, LSD test).

The total concentration of the different families of aromas during the two years studied are shown in Figure 1. It can be difference between acids (A), alcohols (B), esters (C), terpenes and norisoprenoids (D), and miscellaneous (E).

As can be seen in Figure 1, the time of harvest affected the concentration of all the aromatic groups analyzed in both seasons studied. As regards wine acids, they are derived from the grape and yeasts during fermentation and only indirectly affect wine flavor, leading to the production of fatty acid esters [21]. As it can be observed, the MT103 wine showed the highest concentration of acid compounds in both years, but especially in 2019, when the concentration also exceeded that of the Verdejo wine. The MV67 wine had the lowest concentration in 2018, while that made from MV11 had the lowest concentration in 2019. Other authors also mention a significant increase in the concentration of fatty acids when the harvest date was brought forward, such as Sun et al. [22] in the Marechal Foch variety, and Asproudi et al. [10], who explained that the concentration of medium chain fatty acids in Pinot Noir decreased with berry over ripening. On the other hand, the fatty acid concentrations in wines are also dependent on anaerobic growth conditions, must composition, grape cultivar, yeast strain, fermentation temperature, and winemaking practices [14]. However, the same yeast strains, fermentation temperatures, and winemaking practices were used in both years in our study, so the differences between years (Figure 1) were probably due to the must composition being conditioned by the earlier harvest time in 2019 which led to a lower Brix and higher tartaric and malic acid contents.

The second type of volatile compound depicted in Figure 1 is the group of alcohols (Figure 1B), compounds that make a positive contribution to the aroma of wines, enhancing their aromatic complexity [19] when not present in very high concentrations. They are formed mostly from sugars under aerobic conditions, and from amino acids under anaerobic conditions by yeasts through the chemical reduction of the corresponding aldehydes, and are important for the aroma characterization of wines [23].

All the analyzed samples had higher alcohol concentrations in 2019 than in 2018, except the MS30 wine, which had similar concentrations in both years. Sun et al. [22] also observed that the herbaceous C6 alcohols in Marechal Foch wine were the most consistent and showed a more pronounced reduction as a result of late harvest. Nevertheless, the interest in an early harvest, especially in warmer areas, is due to the resulting improvement in balance between the different qualitative components of the berry, particularly the volatile components [10].

In the 2018 season, the MV7 wine had a higher concentration of alcohols than the other wines analyzed, while the wines produced from MV11 and Verdejo had the lowest alcohol content. It is known that the alcohol content of a wine contributes positively to the aroma enhancing its aromatic complexity [24], although concentrations higher than 400 mg L^−1^ have a negative effect due to the appearance of pungent and unpleasant notes [25]. The highest concentration of these compounds was found in MC69 and MV7 wines in 2019, while MS30 produced wines with the lowest alcohol content. The Verdejo wine also had a notably higher concentration of alcohol in this season.

The concentration of alcohol for the all varieties in 2019 was higher than in the 2018 season. In general, temperatures were higher (by 2–6 °C) and rainfall was higher according to data collected in SIAM (Sistema de Información Agraria de Murcia) [26] (data not shown), both of which might have contributed to the higher alcohol concentration. Several authors have reported the effect of climatological conditions on the accumulation of alcohol in wines, which, in our case, could have had effect on grape maturity; for instance, Coelho et al. [27] found that the content of C6 compounds decreased as fruit matured. In our study, the degree of maturity was greater in 2018 (Table 1), thus resulting in less alcohol content than in 2019 (Figure 1B).

During alcoholic fermentation, a great number of esters may be generated as a result of the yeast metabolism, but the most abundant are ethyl esters of organic acids, acetates of fusel alcohols, and ethyl esters of fatty acids [28]. It is generally believed that esters make the greatest contribution to the desirable fermentation bouquet of wine with its characteristic fruity odors [29]. The total concentrations of esters are shown in Figure 1C, where it can be seen that they were slightly higher in 2019 than in 2018. It is known that a higher Brix leads to a substantial decrease in the concentration of esters. According to Kalua et al. [30], after veraison there is less alcohol acyl transferase activity in the berries, and thus, higher ester concentrations in wines obtained from less mature grapes. This would agree with our results for the 2019 wines, a year in which we found a slightly higher quantity of these compounds, increasing the potential for synthesizing esters in grapes that were less mature than those harvested later in the ripening process (2018). In contrast, the MS30 wine showed lower concentrations of these compounds than Verdejo and the rest of the varieties. The results also showed that Verdejo and MC69 wines contained a greater quantity of esters in 2018 and MS33 the least. Verdejo and MC69 wines were again the two ones with the highest ester concentration in 2019, (Figure 1C). According to the results, therefore, the MC69 cross could be considered a good candidate for obtaining a wine with a quality similar to that of a Verdejo wine due to the higher concentrations of these compounds, which would translate into an aromatically fruity wine.

One of the most important groups of volatile compounds in wines are those containing terpenes and norisoprenoids because both are related with quality attributes in wines. They are generally related with floral and citric notes of wine aroma, and are typical of aromatic grape varieties, their importance being related with their low odor threshold [24]. Terpene concentrations are often higher in grapes than in wines, perhaps because the bound terpene glycosides are hydrolyzed to produce volatile aroma compounds or converted into other compounds as a result of molecular rearrangements during fermentation [31]. This might be one of the main reasons for the relatively low quantities found in our study, and only the free terpenes present in the wines were analyzed. The results obtained for this group of compounds are shown in Figure 1D for the two seasons studied. As can be seen, the MT103 wine differed significantly in this respect from the other wines, reaching the highest value in 2018, while the MV67 wine had the highest values in 2019, making this cross the most suitable for obtaining wines with more varietal aromas. In general, the concentrations were slightly higher in all the varieties in 2018 than in 2019, although less so in the case of MV67 and, especially, the MV7 cross, which provided higher concentrations of terpenes than the other crosses and Verdejo. It can be seen that Verdejo wine did not contain higher concentrations than the white crosses, which might mean that this wine would provide lower varietal aromas than some of our new white varieties. In addition, if we take into account that direct sunlight, possibly as a result of high levels of ultraviolet (UV) radiation, enhances both the pre-veraison accumulation of carotenoids and their post-veraison degradation to aroma aroma-activating norisoprenoids [12], so that the different amount of sunlight in different years can influence the obtained results. In this respect, the vineyards received more hours of sunshine in the period studied (April–August) during 2018 than in 2019 (1732 and 1717 h, respectively, according to data from SIAM) [26], which might explain why higher levels of terpenes compounds were synthesized in 2018. Furthermore, the Brix affects grapes, since the concentration of monoterpenes in berries increases during maturation when Brix also increases [19], which is another reason why more terpenes were found in 2018.

Another two compounds found in our wines (a sulfur and a vinyl phenol compound) are both included in the miscellaneous group (Figure 1E). The wine elaborated with MC180 had the highest values, the differences with respect to the other wines analyzed being more significant in 2018, while the wine which had the highest concentration in 2019 was the MV7 wine, followed by MS82 (Figure 1E). Although the concentration of this group of compounds was higher in 2018, it was less pronounced in MV11 and MV7 wines. Once again, the Verdejo wine had the lowest values, which never exceeded 4 mg L^−1^.

### 2.3. Concentration of Individual Aromas in the Different Families of Volatile Compounds

The concentration of individual aromas analyzed in this research are shown in Table 3 and Table 4 for the two years studied. For this research, a quantification method was developed using calibrated curves to evaluate the concentration of all the volatile compounds. In total, 20 compounds were assessed: Three acids, six alcohols, six esters, one sulfur compound, two terpenes, one norisoprenoid, and one vinyl phenol.

#### 2.3.1. Acids

The acids identified in the wines were hexanoic, octanoic, and 9-decenoic acids. While these compounds are not primarily associated with wine quality, their presence plays an important role in the complexity of the aroma [32]. Many of them are thought to be responsible for “green” aromas in grape juice, although they may have less impact in wines [33]. Hexanoic acid is related with cheesy, rancid, and fatty aromas [34]; octanoic acid is related with sweat and cheese aromas [35], and 9-decenoic acid with fatty aromas [34].

The concentrations of all these acids were similar in all the wines analyzed for 2018 (Table 3), ranging from 3 to 7 mg L^−1^, Verdejo wine having the lowest values. By contrast, the concentrations of fatty acids were higher in all the 2019 wines (Table 4), especially as regard hexanoic acid (up to 18 mg L^−1^), while 9-decenoic acid was not identified in Verdejo or MV11 wines during this campaign. These results agree with those of Sánchez-Palomo et al. [36], who found that the wines made from Albillo and Muscat varieties had higher fatty acid concentrations when less ripe grapes were used than the wines made with very mature grapes. Therefore, one of the factors influencing the content of acid compounds in a given wine could be the stage of ripening of the grapes.

It has also been suggested that low impact odorants may act to change the perception of other odorants in a mixture, and, as they may interact synergistically or antagonistically, they may significantly influence perception without being recognized for their OAV (Odour Activity Value) [37]. In addition, the combination of two or more odorants can affect the final aroma, making it more or less intense, and so provide nuances different from those induced by individual odorants [38]. In summary, this kind of aroma was not detected in the sensory tasting because they would have been overwhelmed by other, more intense aromas [36].

#### 2.3.2. Alcohols

A total of six alcohols were identified and quantified in this study (1-propanol, 2-methyl-1-propanol, 3-methyl-1-butanol, 1-hexanol, z-3-hexen-1-ol, and β-phenyl-ethanol) (Table 3 and Table 4). These compounds are formed during alcoholic fermentation and some of them are recognized by their strong and pungent smell and taste and they are related with herbaceous notes. As regard individual compounds, 3-methyl-1-butanol, characterized by burnt or malty notes [39], had the highest concentration in all our studied wines for both years, as found in a similar study carried out by Sánchez-Palomo et al. [3] in Verdejo La Mancha white wines over five consecutive seasons. Another aroma which has an impact in wines is β-phenylethanol, which is synthesized via the Ehrlich pathway through metabolic reactions that involves transamination of the amino acid, L-phenylalanine, and could contribute to the wine aroma with a note of rose [24]. Nonetheless, 1-propanol and 2-methyl-1-propanol have aromas of alcohol, pungent, solvent, or bitter [40], whereas z-3-hexen-1-ol, which was only detected in 2019, has aromas of cut grass and 1-hexanol has notes of vegetables and grass [35].

Table 3 and Table 4 show that the amounts of alcohols detected in our wines was always higher in 2019 than in 2018, which may be explained by the moment of harvest of the grapes, suggesting that it is possible to obtain wines that are more aromatic [41] with a lower alcoholic degree from grapes that are less mature. Pednealut et al. [18] established that the reduction of cis-3-hexenol that occurs during grape ripening may be critical to enhancing wine quality, making this compound a significant marker for harvest decisions because of its herbaceous aroma, which enhances green notes in wine. In our study, (z)-3-hexen-1-ol was not detected in 2018 probably due to the degree of maturity of the grapes used. As demonstrated in previous studies [42,43] the concentration of C6 alcohols decreases as ripening progresses because they are derived from C18 fatty acids via the lipoxygenase pathway and alcohol dehydrogenase, either in situ during grape ripening, or under the oxidative conditions present when fruit is crushed [43,44].

More specifically, in 2018, when all the wines elaborated with the white crosses were compared to Verdejo wine, the highest concentration of 1-propanol and 2-methyl-propanol was found in the MV7 wine. By contrast, the concentration of 3 methyl-1-butanol and β-phenyl ethanol were higher in all the wines made from the crosses, and the same applied to 1-hexanol, except in the case of the MC69 wine, in which the concentration was similar to that of Verdejo wine. 3-methyl-1-butanol may contribute to the complexity of the aroma of wines, although values over 400 mg/L can have a negative effect [45]. In the 2018 season, this compound reached a maximum concentration of 172 mg L^−1^ in the MV7 wine. Furthermore, the positive or negative influence of higher alcohols depends on the type of wine and its aroma [3]. In 2019, Verdejo wine had the highest concentrations of 1-propanol and 3-methyl propanol, and only MC69 wine had a higher concentration of 3-methyl-1-butanol, giving to the wine complexity. By contrast, z-3-hexen-1-ol was detected in all the wines, MC180 and MS82 wines having a higher concentration than Verdejo wine. In the case of 1-hexanol, as in 2018, its concentration was higher in all wines elaborated with the crosses, and β-phenylethanol was only more abundant in MV67 and MV7 wines, giving them rose notes. According to authors such as González-Barreiro et al. [19], 1-hexanol is the result of lipoxygenase activity in the grape and/or aeration of the must. Whatever the case, it fell by 60% as the time before harvesting became longer, probably due to enzyme activity. In addition, Gomez-Míguez et al. [46] found white wines produced from grapes in early maturity exhibited more intense herbaceous notes than those obtained from riper grapes.

#### 2.3.3. Esters

The results obtained for the different esters analyzed in the two seasons are shown in Table 3 and Table 4. The compounds identified, analyzed, and quantified were the following: 3-methyl-1-butanol acetate, ethyl hexanoate, ethyl octanoate, ethyl dodecanoate, ethyl tetradecanoate, and ethyl hexadecanoate. Generally, the esters with the higher concentrations in our wines were 3-methyl-1-butanol acetate, ethyl octanoate, and ethyl hexanoate during both seasons studied. Several descriptors are associated with them; for instance, 3-methyl-1-butanol acetate is related with the positive attribute of “banana” [24], and ethyl hexanoate with apple peel and fruity aromas [47], and finally, ethyl octanoate is related with fruity, banana, or pineapple aromas [36]. Furthermore, it is known that ethyl dodecanoate, ethyl tetradecanoate, and ethyl hexadecanoate impart sweet, waxy, and creamy aromas to the wines [48].

As regards the results obtained in 2018, Table 3 shows that, among the esters analyzed, ethyl octanoate and 3-methyl-1-butanol acetate had the highest concentrations. As mentioned above, these compounds contribute to the fruity character of wines, so Verdejo, MC69, and MT103 wines showed the highest concentration of these compounds and therefore it could be translated in fruity wines. However, this does not mean that these are the only compounds that provide fruit aromas, and, as Robison et al. [14] reported, the aroma of wine is dependent not on a particular compound but on the profile and interactions of the multiple odor-active compounds that are present.

In addition, Verdejo and MC69 wines stood out for their 3-methyl-1-butanol acetate concentration, which would have contributed banana notes. In the case of ethyl dodecanoate, ethyl tetradecanoate, and ethyl hexadecanoate, their concentrations were so low that they probably contributed very little to the complexity of the wines.

On the other hand, taking into account the effect of the moment of harvest in 2019 (Table 4), it seems that this viticultural practice improves the esters composition of wines, since a slight increase in the amounts found in the 2019 wines with respect to 2018 was observed. For instance, early harvesting had a significant effect on the concentrations of ethyl octanoate found in Verdejo, MC69, and MS33 wines, which markedly increased the concentration of these compounds. Moreover, as occurred with the total esters in 2018, due to the results, the high levels shown by the MC69 wine would suggest this cross is a good candidate for obtaining wines with odorant notes similar to those of Verdejo wines. In agreement with our observations, Sánchez-Palomo et al. [36] found that wines obtained from less ripe Albillo and Muscat grapes had a higher ester content than wines made from very mature grapes. The explanation of our results could be that the grapes were harvested sooner in 2019 than in 2018, so this, together with the climatic conditions of 2019 would have increased the ester content of our wines. Furthermore, in Figure S1, the climatic conditions obtained in 2018 and 2019 can be observed.

#### 2.3.4. Terpenes and Norisoprenoids

The linalool, β-damascenone, citronellol, and nerolidol found in the wines analyzed are responsible for varietal aromas [49] and may contribute to improving the perception of the fruity, citrus, and floral aromatic aromas in wines [50]. Linalool and citronellol have aromas of lavender and green lemon, respectively [35], while nerolidol imparts aromas that are sweet and fruity [51]. Other compounds like C13-norisoprenoids (including β-damascenone) may have a great sensorial impact on wines and are normally related with floral and exotic fruit aroma notes [24]. Furthermore, some researchers have suggested that this compound also has an indirect impact on wine aroma by enhancing the fruity notes of the ethyl esters [52].

As can be observed in Table 3, linalool, citronellol, nerolidol were found in trace amounts. However, the norisoprenoid β-damascenone was found in higher concentrations, and presented significant differences in MT103 wine, respective to Verdejo wine, which could suggested that MT103 has more varietal character than Verdejo wine. With respect to individual aromas, β-damascenone was again present at highest concentrations in the MT103 wine, although high concentrations were also found in two more wines; MS30 and MV67. Furthermore, it was not possible to quantify β-damascenone for MV7 in 2018 (Table 3), and MV67 decreased its concentration by half in 2019 (Table 4). On the other hand, Bindon et al. [42] found no significant difference in β-damascenone levels based on the harvest date in Cabernet Sauvignon wines. However, Asproudi et al. [10] observed a statistically significant harvest time-dependent decreasing trend (−15 days, −7 days, and 0 days or full ripeness) for β-damascenone in Pinot Noir and Barbera wines.

Lower concentrations were found in 2019 with respect to the 2018 season (Table 4). Sánchez-Palomo et al. [36] also found lower concentrations of terpenes in Albillo and Muscat wines made from less mature grapes. However, MV67 and MV7 wines presented higher concentrations of terpenes in 2019 for reasons that are not immediately clear, so further studies over more successive years are needed to establish their varietal character or even to study the concentration of bound terpenes. In the present study, terpene concentrations were slightly higher in wines made from mature grapes (2018). This agrees with the findings of Coelho et al. [53], who found that the monoterpenoid content tended to increase with ripening, while other varietal chemicals (C13 norisoprenoids) increased up to maturity and decreased thereafter. Our study, too, suggests that mature grapes provide a higher level of terpenes (Figure 1D, Table 3 and Table 4). Scheneider et al. [54] also affirmed that advanced grape maturity and greater exposure to the sun favor the accumulation of varietal compounds in the berry. The total h of sunshine recorded between the months of veraison and maturation confirm that the grapes would have received 1732 h of sunshine in 2018, compared with 1717 h in 2019, which would explain the higher concentration of terpenes in 2018.

Finally, the Verdejo wine had lower terpene concentrations than the wines made from all the white crosses studied in both seasons, which suggests that the crosses might produce more aromatic wines and be good candidates for producing wines with an aromatic quality similar to or higher than that of Verdejo wine.

#### 2.3.5. Miscellaneous

This group is based on two kinds of compound, one vinyl phenol (4-vinyl-guaiacol) and one sulfur derivate (3-methyl-thio-1-propanol). Vinyl phenols are typically not present in red wines due to the inhibitory action of some tannic substances on these enzymes, but they are the main phenols present in white wines [55]. Most volatile phenols have a negative effect on wine quality; for example, ethyl phenols are responsible for animal and smoky odors, whereas vinyl phenols are associated with strong pharmaceutical odors. However, the compound that we quantified was 4-vinyl-guaiacol, which has aromas of spices like clove or curry [35]. The other miscellaneous compound was 3-methyl-thio-1-propanol, which is related with the catabolic pathway and formed from the amino acid methionine, whose presence depends on the variety of grape [56] and which has descriptors such as cauliflower, cabbage, or potato [57] or aromas related to cooked green beans, wet brush, or green odor [58]. The only “thio” found in this group in our wines was 3-methyl-thio-1-propanol, which, it has been suggested, is formed from the deamination of methionine (sulfur amino acid), followed by decarboxylation and reduction reactions during fermentation [59].

In 2018, the highest concentration of 4-vinyl-guaycol (Table 3) was found in the MV11 wine, while MT103, MS33, and MC180 wines had the lowest values, and Verdejo wine did not show a high concentration of this compound. Slegers et al. [17] also quantified this compound in Vidal IHV (interspecific hybrid *Vitis*) wines, in which it was the main volatile phenol identified, and demonstrated that its odor threshold was 40 μg/L in wines, suggesting that it may affect wine aroma (OAV > 1). As regards 3-methyl-thio-1-propanol, the MC180, MS82, MT103, MV67, and MV7 wines had higher concentrations than Verdejo wine, suggesting cooked vegetable notes, but they were not perceived by the tasters in sensory analysis.

In the 2019 season (Table 4), the concentration of 4-vinyl guaiacol was higher in Verdejo, MV11, MV67, and MV7 wines than in 2018, probably due to the moment of harvest. These results are contrary to those found by Genovese et al. [60], who reported higher levels of 4-vinyl guaiacol in late harvest Fiano wine. By comparison, MC180, MC69, MS30, MS33, MS82, and MT103 wines showed only slight changes in their concentrations. The presence or absence of this compound depends on several pathways; for example, according to Chatonnet et al. [61], 4-vinyl guaiacol in white wines is formed by yeasts through the decarboxylation of ferulic acid, while Ugliano et al. [62], also in Fiano white wine, suggested it was formed as a result of glycosides hydrolysis, a pathway that would appear to merit further investigation. Finally, Quian et al. [63] found that 4-vinyl guaiacol with its spicy, clove character, was generated through the breakdown of lignin.

In 2019, the concentrations of sulfur compounds were lower than in 2018 for all the varieties studied, also would have been affected by the time of harvest, which would have improved the sensory quality of wines.

### 2.4. Sensory Analysis

A panel of professional tasters characterize aromas in these new varieties from 2016 until 2019 as can be seen in our previous research [64] and this paper. In all of the studies made by our research group, with respect to the aroma assessment in these new varieties, it can be seen how the aromatic characterization of the wines was made according to the optimum moment of harvest. It has been demonstrated that the harvest date (directly related with the degree of maturity of the grape) could affect aroma characteristics in wines, and therefore be associated with their final quality.

**Figure 2:**
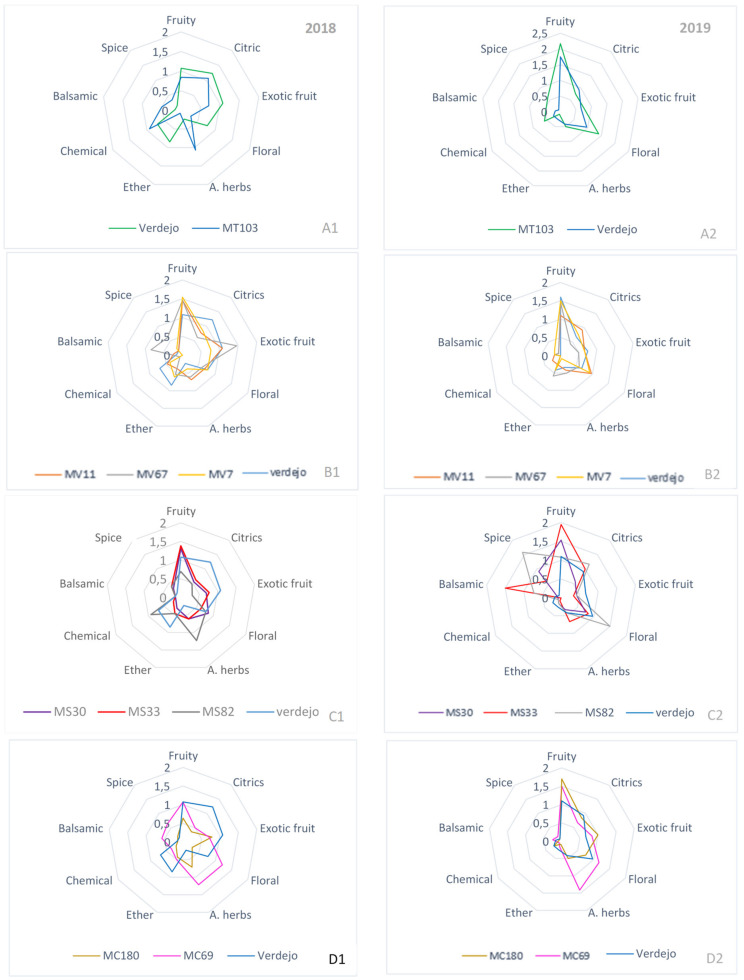
Sensory analysis of 2018 (**A1**–**D1**) and 2019 wines (**A2**–**D2**). (**A**) shows Tempranillo cross with Monastrell. (**B**) encompasses the Verdejo family. (**C**) shows the different crosses of Monastrell x Syrah. (**D**) indicates the crosses of Monastrell x Cabernet Sauvignon. The aroma characteristics have relative standard deviation of below 0.05 (RSD < 0.05).

Figure 2 represents the spider graphics corresponding to the aroma tasting results for the two years studied. All descriptors corresponding to each series can be seen in Table S2. As can be seen, the profiles corresponding to the same wines differed between years. The results are divided into four groups, taking into account their parental and comparing the wines with Verdejo wine. The first aim was to ascertain whether the moment of harvest had influenced the result, while bearing in mind that the grapes in 2018 were collected with a higher Brix than in 2019. In addition, in Tables S3 and S4, the Duncan post-hoc test can be seen to gain more information.

The second aim was to compare the sensory analysis between Verdejo wine and the wines obtained from the new crosses, in order to determine whether any of the new varieties could induce similar or better organoleptic characteristics than Verdejo in the resulting wines. The results differed between years, and in 2018 Verdejo wine presented pleasant citric notes and some aromas from the ether series, for example, stearin aroma and ripe banana. The same wine also had some aromas from the chemical series which recalled artichoke (not very common in this variety), probably due to the Brix in this year. By contrast, in 2019 the wine aromas showed more fruity notes, like green apple, tropical fruit (pineapple), and floral (jasmine, orange blossom) aromas. These results agree with the those of Sánchez-Palomo et al. [3], who found the same characteristics in Verdejo white wines from La Mancha.

In Figure 2A, Verdejo wine is compared with the MT103 wine in both years (1 and 2, respectively). In 2018, the MT103 wine was considered by tasters to be a citric wine, with notes of aromatic herbs and some aromas of the chemical series, like alcohol or solvent, and less aromatic than Verdejo (Figure 2A1). However, in 2019, this wine was defined as being more aromatically complex, with stronger notes of fruits (apricot and peach), which may have been related with the ethyl ester concentrations resulting from the earlier harvest and lower Brix than in 2018. Furthermore, lemon or orange citric notes and some floral characteristics (jasmine) (Figure 2A2) added to the improvement in the aromatic composition compared with Verdejo during this last year.

Among the Verdejo group wines (Figure 2B1), MV11, MV67, and MV7 wines were considered more aromatic than Verdejo wine in 2018. Wines from the crosses were considered as fruity (peach and some red stone fruits) and balsamic, with notes of pine tree and exotic fruit (pineapple and lychee aromas). On the other hand, the Verdejo crosses (Figure 2B2) in 2019 were favorably accepted, with stronger fruity notes than the 2018 wines; the MV7 wine, for example, had notes of apricot, the MV67 wine peach aromas, and the MV11 wine quince aromas and pleasant citric notes (orange and lemon). Aromas reminiscent of hay or herbaceous plants were also observed in the Verdejo crosses.

Figure 2C1 shows the group of Syrah wines. In 2018, MS30 and MS33 wines were characterized as having aromas of fruit and exotic fruits like peach and apricot, while the MS82 wine showed more characteristics of aromatic herbs and some chemical aromas (sulfur or solvent notes). In this year, Verdejo still had marked floral and citrus notes although the MS30 and MS33 wines stood out for their fruity notes. Again in 2019, Syrah crosses (Figure 2C2) were characterized by the fruity wines they produced, especially MS33 with notes of young red fruit, some balsamic notes, and fresh herb with lemon leaf, all more intense than in Verdejo. The MS82 wine had aromas from the spice series (menthol), floral notes (citronella), some notes of green apple, and eucalyptus aromas and the MS30 wine had more aromas of green apple, lemon, pineapple, and fresh herbs than Verdejo wine.

Finally, the wines obtained from the Cabernet crosses are shown in Figure 2D. In the 2018 season (D1), the wine from MC69 was considered as floral (white flowers) with pleasant notes of aromatic herbs like mint or peppermint, and scored better than the Verdejo wines for their organoleptic characteristics, while the MC180 wine showed the same notes but less pronounced, probably due to the maturity of the grape. MC180 also had peach and lychee aromas and generally had more subtle aromas than Verdejo. As regards the data obtained for 2019 (Figure 2D2), the mint and peppermint aromas were enhanced, as were white floral aromas (jasmine) in the MC69 wine and, probably due to the later harvest, it also had notes of red fruit (apricot), lychee, and sweet spices like ginger. MC180 was also valued positively for being a well-structured aromatic wine with aromas recalling peach, passion fruit, peppermint, some citric notes, and ginger and spicy notes. In summary, as can be seen in Figure 2D2 the aromatic quality of the wines obtained from these crosses was notably superior to that of Verdejo wine.

According to the data outlined in Figure 2 A2, B2, C2, and D2, and the views of the tasters, it could be seen that wines from 2019 were fresher and more pleasant than those from 2018, very probably as a result of the earlier harvest time in 2019.

### 2.5. Two-Way ANOVA Analysis

The aromatic composition is shown in Table 5, in which an analysis of variance shows the different groups of aromatic compounds in wines as a function of variety and year.

As can be seen in Table 5, there is a great variability for different samples in terms of the concentrations obtained in the different families of volatile compounds. Compared with Verdejo, a higher concentration of acids was obtained in the new varieties as is the case with terpenes and miscellaneous, less in MV7 in terpene concentration. Contrarily, MV7 is surpassed in alcohol concentration (concentration is beginning to be critical) and MV69. Verdejo obtained a higher ester concentration.

On the other hand, in the vintage (and therefore at the time of harvest) the effect is clearly seen; in 2019 it obtained a higher concentration of all the families of volatile compounds except terpenes, which are synthesized during maturation. As can be seen, there is a clear interaction between the year and the variety, therefore it is clear evidence that the answer will be one or the other depending on the variety.

### 2.6. Clustered Heat Map

**Figure 3:**
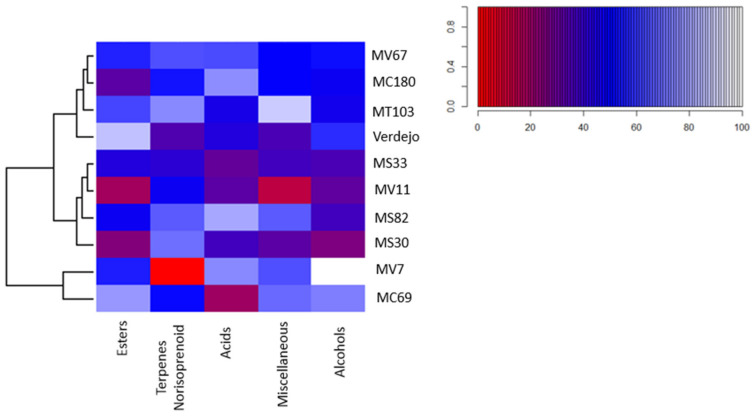
Heat map correlations for the different aromatic families with elements sorted according to a cluster analysis of the crosses and Verdejo. In brief, white colors indicate positive correlations, red colors indicate negative correlations, and blue colors indicate intermediate correlations.

The diversity of the individual crosses with the principal aromatic groups was visualized by hierarchical clustering and a heat map (Figure 3). Heat maps are ubiquitous in the genomics literature and are very useful for visualizing the measurements by means of a subset of rows representing all the samples. Each row depicts the main group for the individual varieties.

With the distance between samples computed, clustering algorithms are needed to join them into groups. The clustering of white crosses and Verdejo with the groups of aromtic compounds had a correlation coefficient of 0.81 when 1 is the maximum, and mainly showed three different groups, with wines of Verdejo, MT103, MC180, and MV67 grouped closely; while MS33, MV11, MS82, and MS30 crosses grouped in another package; and a final group formed by MV7 and MC69. These three groups conform the hierarchy.

In addition, our results showed how esters, followed by alcohols, were the aromatic groups with most weight in the heat map analysis in Verdejo wine, while MT103 was characterized by its acidity, and terpene and norisoprenoid groups; MC180 by its miscellaneous aromas, and finally MV67 by the same group but also terpenes and norisoprenoids. It is necessary to emphasize the importance of the weight of each aromatic group. According to Vilanova et al. [65], wine quality is closely related with overall aroma and therefore with the wine volatile composition responsible for these aromas. Our study of the volatile composition of wines and their sensory analysis will contribute to improving knowledge of what makes a quality wine.

Finally, as Ghaste et al. [66] suggest, the database built in this study could be applied in the identification of aromatic quality of wines made with our crosses in future studies, in order to systematically profile the different wines, and elaborate wines with the best aromatic qualities using genotypes available in ampelographic collections.

## 3. Materials and Methods

### 3.1. Experimental Design

#### 3.1.1. Chemicals

1 propanol, 2-methyl-1-propanol, 3-methyl-1-butanol-acetate, 3-methyl-1-butanol, ethyl hexanoate, 1-hexanol, Z-3-hexen-1-ol, ethyl octanoate, linalool, ethyl decanoate, 3-methyl-thio-1-propanol, β-damascenone, citronellol, ethyl dodecanoate, hexanoic acid, β-phenyl ethanol, nerolidol, ethyl tetradecanoate, octanoic acid, 4-vynil guaiacol, ethyl hexadecanoate, 9-decenoic acid, and 2-octanol were supplied by Sigma Aldrich (Madrid, Spain). Ultrapure water from a Milli-Q system (Millipore Corp., Bedford, MA). NaCl, NaOH, and ethanol 96% were from Panreac (Barcelona, Spain). Tartaric acid from Laffort (France) was used to adjust model wine.

#### 3.1.2. Vineyards

The study was carried out in an experimental vineyard, “El Chaparral,” located in Bullas (Murcia, Spain), latitude 38.11179 and longitude −1.6808. Seeds from different crosses between Monastrell (M) and Tempranillo (T), Syrah (S), Verdejo (V), Cabernet Sauvignon (C) (named MC180, MC69, MS30, MS33, MS82, MT103, MV11, MV67, and MV7) were planted between 2007 and 2012 and the parentals in 1997. The plantation framework was 3 m × 1.25 m with a density of 2670 vines/ha. Twenty plants per crossing were planted in a trellis system. The vines received deficit drip irrigation, with a maximum of 0.665 m^3^/year per plant, depending on annual rainfall.

The climate of the growing area is categorized as semi-arid continental, and the soil as loamy sand. The weather conditions for the two years studied led to the harvest being later in 2018, when the herbaceous-growth period was wet in April, May, and June, and very sunny. In 2019, the herbaceous-growth period was slightly warmer and drier, with a significant amount of rain only falling in April, although it was also very sunny.

#### 3.1.3. Winemaking

The grapes were harvested manually at different moments depending on the year. In 2018 the grapes had more than 21 Brix, and, in order to study whether the aroma extracted from the different families was affected by the same, the grapes in 2019 were harvested with no more than 20 Brix. In both cases, the content in malic acids was taken into account (close to 2 g L^−1^). In the winery, the wines were elaborated in 50 L steel tanks and the elaboration volume was 25 L by microvinification. Highlight the acidification of the Verdejo, MC180, MS33, and MV11 musts in the 2018 campaign due to their low values and the need to have approximate values in the samples. Prior to alcohol fermentation, the wines underwent a racked and pre-fermentative maceration of 3 h at 10 degrees. Neutral aroma yeasts (Zymaflore Spark Sacharomyces cerevisiae) were used. The reducing sugars were measured with a Cetlab 600, (Microdom, Taverny, France) while the density was measured with a PROTON aerometer. When reducing sugars measured <0.2 g L^−1^ and the density was <1.0 g mL^−1^, the alcoholic fermentation was terminated. The fermentation temperature begins at 18 °C and ends at 22 °C. We rely on this control temperature to favor varietal aromas over fermentation aromas. Then, pectolytic enzymes were added (Lafazym^®^CL, Moût et Vin) for clarification and the wines were kept in 15 L bag in box recipients and stored in a conditioned room maintained at 8–10 °C. The samples were analyzed in triplicate at the end of alcoholic fermentation.

#### 3.1.4. Physicochemical Determinations in Grapes and Wines

Several analyses were carried out when the grapes reached the winery: The must parameters were measured with an Atago RX-5000X refractometer (Atago CO., LTD, Tokyo, Japan) for Brix and density, and malic acid was measured with a CETLAB 600 (Microdom, Taverny, France), while pH and total acidity were measured with a Schott, alpha plus TA20 (SCHOTT-GERÄTE GmbH, Mainz, Germany). When the wines had finished alcoholic fermentation, total acidity (g L^−1^) and pH levels were quantified with a Metrohm 824 (Metrohm AG, Herisau, Switzerland) using a glass electrode (6.0233.100; Metrohm, Herisau, Switzerland). Finally, the alcohol percentage was measured (*v*/*v*) with an Anton Paar SP-1 M Wine Alcolyzer (Anton Paar GmbH, Graz, Austria).

#### 3.1.5. Calibration Curves

Calibration curves were obtained using a synthetic wine model (3 g tartaric acid, 10% ethanol, and pH adjusted to 3.5 with NaOH) containing the compounds of interest at five increasing concentrations, all within the ranges normally found in wines. The limits of detection and quantification for the different compounds analyzed are shown in Table S1. The methodology used was the same as for the analysis of volatile compounds in the different samples (See Section 3.1.6)

#### 3.1.6. Sample Preparation

The technique used in this study to quantify the precise concentration of aroma in a wine was headspace solid-phase microextraction (SPME), which has previously been applied to the analysis of volatile compounds in many wines because of its ease of use, good reproducibility, and lack of a need for large samples or solvents of any kind [67]. The fiber coating is the key to SPME, because different fibers have different selection and extractive efficiencies for volatile compounds [68]. In this study we used the PDMS/DVB/CAR fiber coating, as several authors have obtained good results with it; for example Gomez-Plaza et al. [69] used it to study the effect of two elicitors, benzothiadiaole and methyl jasmonate, on the volatile compound composition of Monastrell grapes and wines, while Gil-Muñoz et al. [50] used it to study the effect of using purified grape pomace as fining agent on the volatile composition of Monastrell wines. Liu et al. [68] used it in the characterization of the key aroma compounds in Proso Millet wine and, finally, Arcari et al. [70] studied the volatile composition of Merlot red wine and its contribution to the aroma using, among others, this fiber. The chromatograms obtained can be seen in Figure S2, where the overlap of the Verdejo aromatic profile is seen together with a Monastrell, MC69 cross.

Ten milliliters of wine were added to 20 mL vials with a magnetic screw top and polytetrafluoroethylene (PTFE)-lined septum, and then 25 μL of 2-octanol as internal standard (100 ppm) (Sigma-Aldrich, Madrid, Spain) and 3 g of NaCl were added. A Gerstel auto-sampling device (Gerstel GmbH&Co. KG, Mellinghofen, Germany) was used. Gas chromatographic studies were carried out on an HP 7890B gas chromatograph (GC) system coupled to an HP 5977A quadrupole mass spectrometer (Agilent Technologies, Palo Alto, CA, USA), following the method described by Gómez-Plaza et al. [69] and Moreno-Olivares et al. [64]. The injector was run in splitless mode for 0.75 min with a 2 mm inner diameter non-deactivated direct liner for the SPME (Agilent Technologies) and a desorption time of 5 min. A divinylbezene (DVB)-carboxen (CAR)-polydimethylsiloxane (PDMS) fiber (Supelco, Bellefonte, PA, USA) and a DB-WAXetr capillary column (30 m × 250 µm, 0.25 μm film thickness; Agilent Technologies) were used, with helium 8.0 as gas (Abelló Linde SA, Barcelona Spain) and a column head pressure of 8 psi. The oven temperature was maintained at 40 °C for 0.5 min, raised to 260 °C at 4 °C min^−1^ and then held at that temperature for 5 min. The mass spectrometer was operated in electron ionization mode at 70 eV with the detector in scan mode (mass range 20–350 amu) and the transfer line to the mass spectrometer system was maintained at 230 °C. Peaks were identified with the mass library (Wiley Registry 6.0; Wiley, Chichester, UK). The positively identified compounds were quantitatively analyzed by total ion current using the calibration curves proposed for each sample. All samples were analyzed in triplicate.

#### 3.1.7. Sensory Analysis

The sensory analysis was made by a panel of qualified tasters formed by 12 professionals, who completed a technical tasting sheet. The training took place over eight weeks. During the training, panelists tasted different aroma standards and rated the different attributes which span different groups of aromas (fruity, citrus, exotic fruits, flowers, aromatic herbs, ethereal series, chemical series, balsamic series, and spicy series). Tasters used a scale from 0 to 5 to rate the specific aroma intensity. In the first session the judges were asked to provide descriptors in a series of typical aromas of white wines (see Table S2 sensorial analysis from Supplementary Materials). In the next session, each specific aroma was studied, and an attempt was made to memorize them. Finally, in the remaining weeks of training, tests were carried out in order to establish knowledge. In this way, the judges managed to obtain uniformity in the results.

Although the main objective was to the focus on aroma phase, the tasting took into account the three phases: Visual, olfactory, and gustatory (visual and gustatory phases are not shown in the results).

#### 3.1.8. Data Processing and Statistical Analysis

Significant differences among wines and for each variable were assessed by analysis of variance. A least significant difference test was used to separate the means (*p* < 0.05) by LSD analysis. The analysis was conducted using R Studio 3.6.2 and the cluster analysis and heat map visualization of the compounds were carried with R Studio 1.1.453 (both from Boston, MA). Furthermore, the effect of variety and year on the aromatic composition was analyzed by two-way ANOVA with interactions using Statgraphics 5.0 Plus.

## 4. Conclusions

In this article, the foreseeable effects of climate change and corresponding moment of harvest on the volatile composition of white wines made from Monastrell crosses were studied. The wines were compared with a Verdejo wine, considered to be a Spanish quality white wine. The harvest time was considered to be an important factor since the total concentrations of acids, alcohols, and esters decrease as the maturity of the grapes increases. If the grapes are harvested with a lower ºBrix (by bringing forward the harvest time), the total amount of these aromatic groups increases. However, the concentration of terpenes, norisoprenoids, and miscellaneous fall as the grapes ripen. According to the data analyzed, advancing the harvest time provides grapes with a lower ºBrix which could help to obtain grapes which could be organoleptically more suitable for making quality white wines, since a pH lower than 3.5 is obtained and a concentration of malic acid more than 2 g L^−1^, especially if the MC69 cross is used. The time of harvest strongly influenced aromatic profile of wines, affecting their overall aroma characteristics, which is correlated with the final quality. The results obtained suggest that the cultivation of these new varieties could be an option for obtaining new varieties adapted to the climatological characteristics of this Mediterranean area, especially in light of predicted climate change.

Finally, characterization of the new Monastrell white crosses showed that the aromatic composition of some of them, such as MT103, MC180, and MC69, is very similar to and even better than that of Verdejo wine. In short, together with the previously published data [64], this study shows that these new varieties are well-adapted to the new edaphoclimatic scenario of south-eastern Spain and are suitable for obtaining wines with good organoleptic characteristics. However, it is also necessary to take into account that comparing samples from different harvest times but from different years cannot be an indicator of only the harvest time. It would be necessary to conduct a comparison in different dates of harvest in the same year. For this, it is necessary that the new plantations begin to produce grapes to have more material to study.

## Figures and Tables

**Table 1:** Analytical parameters in must at harvest and in wine at the end of alcoholic fermentation in 2018 season.

	Verdejo	MC180	MC69	MS30	MS33	MS82	MT103	MV11	MV67	MV7	*p*-Value
**HARVEST DATE**	27 Aug*	29 Aug	29 Aug	03 Sep	28 Aug	28 Aug	29 Aug	03 Sep	30 Aug	29 Aug	
**MUST**											
**Brix**	21.73 d	22.45 a	22.30 b	22.51 a	22.24 b	22.51 a	22.10 c	20.91 e	22.54 a	22.23 bc	***
**Total acidity**	3.25 h	3.86 g	5.95 b	6.14 a	3.25 h	4.57 e	5.14 c	3.92 g	4.96 d	4.35 f	***
**pH**	3.73 a	3.71 ab	3.46 d	3.38 e	3.66 b	3.35 e	3.37 e	3.73 a	3.55 c	3.53 c	***
**Tartaric acid**	4.21 h	4.08 i	4.75 g	6.21 a	5.06 e	6.12 b	5.83 c	4.21 h	5.34 d	4.94 f	***
**Malic acid**	2.30 e	2.86 d	3.77 a	3.12 b	2.11 g	2.21 f	1.91 h	3.05 b	2.94 c	2.08 g	***
**WINE**											
**Alcohol %**	12.91 h	13.46 g	13.68 cd	13.62 e	13.76 a	13.71 bc	13.65 de	12.73 i	13.75 ab	13.57 f	***
**pH**	3.25 c	3.29 bc	3.53 a	3.29 bc	3.15 e	3.15 e	3.15 e	3.24 cd	3.33 b	3.18 de	***
**Total acidity**	7.03 e	7.63 c	7.03 e	6.86 f	6.72 g	7.93 a	7.21 d	6.64 h	6.52 i	7.85 b	***

The relative standard derivation is less than 0.05 (RSD < 0.05) in all the analyses. Total acidity, tartaric acid and malic acid are measured in g L^−1^. * Aug: August; Sep: September.

**Table 2:** Analytical parameters in must at harvest and in wine at the end of alcoholic fermentation in 2019 season.

	Verdejo	MC180	MC69	MS30	MS33	MS82	MT103	MV11	MV67	MV7	*p*-Value
**HARVEST**	20 Aug	26 Aug	22 Aug	26 Aug	20 Aug	26 Aug	20 Aug	26 Aug	22 Aug	26 Aug	
**MUST**											
**Brix**	19.73 b	18.24 f	19.34 d	17.33 h	19.52 c	17.74 g	19.91 a	19.84 a	19.50 c	19.15 e	***
**Total acidity**	5.26 f	5.56 e	7.38 a	7.08 b	4.54 h	5.84 d	6.25 c	5.05 g	5.93 d	5.26 f	***
**pH**	3.54 a	3.42 bc	3.34 cd	3.15 e	3.45 abc	3.34 cd	3.25 de	3.53 ab	3.46 abc	3.44 abc	***
**Tartaric acid**	5.13 ef	4.86 g	5.08 f	6.71 a	5.22 e	5.77 c	6.09 b	4.54 h	5.60 d	5.67 cd	***
**Malic acid**	3.26 b	3.21 b	4.22 a	2.54 e	1.96 h	2.31 f	2.77 d	2.91 c	2.88 cd	2.16 g	***
**WINE**											
**Alcohol %**	11.57 de	10.62 f	11.46 e	10.04 h	11.90 b	10.44 g	12.16 a	12.03 ab	11.71 c	11.64 cd	***
**pH**	3.44 a	3.42 b	3.40 bc	3.09 h	3.17 g	3.25 f	3.23 f	3.33 d	3.39 c	3.29 e	***
**Total acidity**	5.64 g	5.66 fg	6.90 b	7.03 a	6.14 d	5.77 ef	6.12 d	6.47 c	5.84 e	5.69 fg	***

The relative standard derivation is less than 0.05 (RSD < 0.05) in all the analyses. Total acidity, tartaric acid and malic acid are measured in g L^−1^. * Aug: August.

**Table 3:** Individual aromatic concentrations in wines (expressed as mgL^−1^) at the end of alcoholic fermentation in 2018.

	IK^2^	VERDEJO	MC180	MC69	MS30	MS33	MS82	MT103	MV11	MV67	MV7	*p*-Value^4^
**ACIDS**												
**Hexanoic acid**	1876	4.05 ± 0.17 d^1^	4.05 ± 0.17 d	5.65 ± 0.13 a	4.85 ± 0.29 bc	4.05 ± 0.09 d	4.74 ± 0.04 c	5.48 ± 0.32 a	3.80 ± 0.13 d	4.75 ± 0.05c	5.15 ± 0.16 b	***
**Octanoic acid**	2092	3.22 ± 0.38 f	4.50 ± 0.11 de	5.78 ± 0.15 c	4.65 ± 0.14 d	4.18 ± 0.12 de	5.93 ± 0.45 c	7.84 ± 0.57 a	4.04 ± 0.08 e	nd^3^	6.62 ± 0.38 b	***
**9-decenoic acid**	2386	4.31 ± 0.07 e	5.57 ± 0.20 bc	5.61 ± 0.10 bc	5.84 ± 0.47 b	5.49 ± 0.10 bc	5.53 ± 0.17 bc	6.23 ± 0.34 a	5.73 ± 0.18 b	4.72 ± 0.17 d	5.24 ± 0.10 c	***
**ALCOHOLS**												
**1-propanol**	1059	19.85 ± 0.93 b	12.45 ± 2.72 bc	14.60 ± 1.54 bc	12.74 ± 1.72 bc	8.64 ± 0.62 c	11.34 ± 1.55 c	10.63 ± 1.85 c	7.72 ± 0.62 c	15.27 ± 5.84 bc	28.71 ± 6.50 a	**
**2-methyl-1-propanol**	1123	37.71 ± 2.95 b	33.77 ± 1.32 bcd	22.18 ± 0.63 e	32.50 ± 3.87 cd	31.10 ± 2.61 d	15.00 ± 0.63 f	32.29 ± 4.80 cd	20.56 ± 2.40 e	36.73 ± 3.12 bc	44.22 ± 3.44 a	***
**3-methyl-1-butanol**	1233	82.07 ± 0.78 f	155.63 ± 5.46 ab	115.54 ± 6.41 de	135.10 ± 6.32 cd	128.72 ± 6.23 cde	134.63 ± 6.01 cd	130.13 ± 7.27 cd	109.65 ± 8.45 e	143.87 ± 4.27 bc	172.47 ± 3.80 a	***
**1-hexanol**	1371	1.05 ± 0.26d	1.94 ± 0.10 b	1.22 ± 0.06 d	2.17 ± 0.21 b	1.54 ± 0.06 c	1.95 ± 0.12 b	2.19 ± 0.08 b	1.96 ± 0.11 b	2.08 ± 0.22 b	2.46 ± 0.17 a	***
**z-3-hexen-1-ol**	1378	nd	nd	nd	nd	nd	nd	nd	nd	nd	nd	
**β-phenyl-ethanol**	1931	29.52 ± 0.71 fg	39.78 ± 0.54 c	36.45 ± 0.37 cde	38.68 ± 1.33 cd	32.51 ± 0.69 ef	50.44 ± 5.86 a	47.48 ± 3.05 ab	26.59 ± 1.72 g	35.14 ± 0.56 de	45.43 ± 3.53 b	***
**ESTERS**												
**3-methyl-1-butanol-acetate**	1141	2.48 ± 0.19 a	1.17 ± 0.11 de	1.84 ± 0.10 b	1.71 ± 0.04 b	0.99 ± 0.07 ef	1.57 ± 0.11 bc	1.1 ± 0.11 6de	0.81 ± 0.04 f	1.52 ± 0.17 bc	1.33 ± 0.49 cd	***
**Ethyl-hexanoate**	1254	0.38 ± 0.01 f	0.57 ± 0.01 cd	0.66 ± 0.02 bc	0.60±0.03 bcd	0.51 ± 0.02 de	0.64 ± 0.008 bc	0.91 ± 0.10 a	0.47 ± 0.11 ef	0.69 ± 0.01 b	0.65 ± 0.10 bc	***
**Ethyl-octanoate**	1458	1.67 ± 0.08 a	0.86 ± 0.05 d	1.74 ± 0.04 a	0.90 ± 0.02 d	0.86 ± 0.06 d	1.09 ± 0.09 c	1.32 ± 0.04 b	1.10 ± 0.06 c	1.65 ± 0.15 a	1.41 ± 0.10 b	***
**Ethyl-dodecanoate**	1857	0.15 ± 0.01d	0.63 ± 0.12 a	0.23 ± 0.01 cd	0.45 ± 0.04 b	0.17 ± 0.01 cd	0.24 ± 0.007 c	0.57 ± 0.07 a	0.240.004 ±c	0.37 ± 0.09 b	0.41 ± 0.02 b	***
**Ethyl-tetradecanoate**	2037	0.011 ± 0.001 de	0.022 ± 0.004 b	0.018 ± 0.0003 bc	0.022 ± 0.004 b	0.012 ± 0.003 de	0.016 ± 0.003 bcd	0.015 ± 0.004 cde	0.011 ± 0.003 de	0.010 ± 0.003 e	0.029 ± 0.01 a	***
**Ethyl-hexadecanoate**	2264	0.011 ± 0.001 e	0.022 ± 0.0004 c	0.019 ± 0.001 c	0.028 ± 0.001 ab	0.015 ± 0.002 d	0.021 ± 0.002 c	0.019 ± 0.002 c	0.012 ± 0.0008 de	0.029 ± 0.003 a	0.026 ± 0.001 b	***
**TERPENES AND NORISOPRENOIDS**												
**Linalool**	1542	0.005 ± 0.0003 g	0.007 ± 0.0005 de	0.017 ± 0.0002 b	0.008 ± 0.0005 c	0.005 ± 0.0006 fg	0.007 ± 0.0003 de	0.006 ± 0.001ef	0.023 ± 0.001 a	0.008 ± 0.0001 cd	0.004 ± 0.001 g	***
**β-damascenone**	1755	2.603 ± 0.07 e	3.461 ± 0.49 bc	3.123 ± 0.09 cd	3.740 ± 0.42 ab	2.717 ± 0.24 de	3.871 ± 0.06 ab	4.006 ± 0.21 a	2.924 ± 0.28 de	2.946 ± 0.13 de	nd	***
**Citronellol**	1785	0.008	0.009	0.009	0.009	0.008	0.010	0.008	0.009	0.008	0.008	ns
**Nerolidol**	2071	0.008 ± 0.001 d	0.017 ± 0.004 ab	0.012 ± 0.001 cd	0.014 ± 0.002 bc	0.012 ± 0.001 cd	0.019 ± 0.01 a	0.015 ± 0.001 abc	0.015 ± 0.004 abc	nd	0.017 ± 0.003 ab	***
**MISCELLANEOUS**												
**4-vinyl-guaaiacol**	2262	0.28 ± 0.01 bc	0.26 ± 0.03 bc	0.34 ± 0.01 abc	0.27 ± 0.01 bc	0.25 ± 0.02 c	0.29 ± 0.05 bc	0.25 ± 0.03 c	0.42 ± 0.03 a	0.35 ± 0.17 ab	0.31 ± 0.04 bc	*
**3-methyl-thio-1-propanol**	1724	3.7 ± 0.20 cd	6.5 ± 0.29 a	2.9 ± 0.19 e	3.6 ± 0.62 cd	3.6 ± 0.30 d	5.2 ± 0.33 b	5.1 ± 0.44 b	2.6 ± 0.30 e	4.3 ± 0.35 c	4.1 ± 0.54 cd	***

^1^ Different letters in the same row point to significant differences (ANOVA, LSD test). ^2^ IK indicates Kovat’s retention index. ^3^ nd, aroma not detected. ^4^*p*-value < 0.05.

**Table 4:** Individual aromatic concentrations in wines (expressed as mgL^-1^) at the end of alcoholic fermentation in 2019.

	IK^2^	VERDEJO	MC180	MC69	MS30	MS33	MS82	MT103	MV11	MV67	MV7	*p*-Value^4^
**ACIDS**												
**Hexanoic acid**	1876	16.27 ± 2.84 ab^1^	13.41 ± 1.29 bcd	16.79 ± 2.98 a	11.66 ± 0.28 d	13.03 ± 0.54 bcd	15.40 ± 2.28 abc	18.04 ± 1.96 a	12.45 ± 0.51 cd	16.08 ± 1.16 ab	16.02 ± 2.21 ab	**
**Octanoic acid**	2092	10.48 ± 2.94 bc	11.32 ± 2.22 bc	13.16 ± 3.57 ab	5.26 ± 0.24d	7.51 ± 0.77 cd	12.64 ± 3.62 ab	16.38 ± 2.85 a	3.81 ± 0.47 d	13.70 ± 2.88 ab	11.46 ± 2.98 bc	***
**9-decenoic acid**	2386	nd^3^	4.72 ± 0.13 c	4.91 ± 0.62 bc	4.93 ± 0.02 bc	4.83 ± 0.11 bc	6.34 ± 0.61 a	4.88 ± 0.33 bc	nd	4.46 ± 0.10 c	5.30 ± 0.42 b	***
**ALCOHOLS**												
**1-propanol**	1059	23.34 ± 5.14 a	7.88 ± 1.55 d	14.04 ± 3.36 bc	6.12 ± 0.17 d	9.33 ± 0.23 cd	7.96 ± 0.65 d	17.81 ± 2.95 ab	6.12 ± 1.65 d	9.66 ± 1.04 cd	10.44 ± 1.87 cd	***
**2-methyl-1-propanol**	1123	45.12 ± 7.89 a	23.13 ± 3.42 cd	37.37 ± 9.54 ab	21.07 ± 0.52 d	23.79 ± 2.62 cd	22.16 ± 3.54 cd	33.76 ± 3.05 abc	30.89 ± 3.82 bcd	24.83 ± 2.18 cd	33.59 ± 4.11 abc	**
**3-methyl-1-butanol**	1233	228.46 ± 13.15 b	176.78 ± 9.63 cd	273.29 ± 8.30 a	141.45 ± 2.06 f	168.07 ± 11.80 cde	155.12 ± 6.32 ef	165.30 ± 2.76 de	185.69 ± 9.63 c	173.49 ± 3.26 cde	216.08 ± 10.37 b	***
**1-hexanol**	1371	2.33 ± 0.77 g	4.97 ± 0.47 cde	5.27 ± 0.97 cd	7.05 ± 0.13 a	3.89 ± 0.26 f	5.76 ± 0.39 bc	4.36 ± 0.79 ef	4.48 ± 0.19 def	4.10 ± 0.11 ef	6.17 ± 0.3 ab	***
**z-3-hexen-1-ol**	1378	0.49 ± 0.08 c	1.07 ± 0.15 b	0.54 ± 0.19 c	0.69 ± 0.004 c	0.61 ± 0.02 c	1.61 ± 0.27 a	0.64 ± 0.04 c	0.61 ± 0.28 c	0.47 ± 0.22 c	0.83 ± 0.35 bc	***
**β-phenyl-ethanol**	1931	47.60 ± 2.14 cd	47.53 ± 5.91 cd	46.53 ± 1.81 cd	35.22 ± 0.55 d	51.31 ± 4.44 c	58.58 ± 2.37 bc	61.47 ± 5.46 abc	54.93 ± 1.76 bc	77.05 ± 1.90 a	70.33 ± 2.96 ab	***
**ESTERS**												
**3-methyl-1-butanol-acetate**	1141	3.68 ± 0.56 a	1.04 ± 0.33 ef	2.97 ± 0.37 b	0.69 ± 0.01 fg	1.08 ± 0.14 ef	1.33 ± 0.19 de	2.23 ± 0.01 c	0.31 ± 0.1 g	1.83 ± 0.25 cd	1.29 ± 1.79 e	***
**Ethyl-hexanoate**	1254	1.10 ± 0.18 b	0.99 ± 0.02 b	1.23 ± 0.35 b	0.63 ± 0.01 b	1.05 ± 0.05b	1.06 ± 0.08 b	2.22 ± 1.08 a	0.70 ± 0.02 b	1.18 ± 0.02 b	1.20 ± 0.4 b	**
**Ethyl-octanoate**	1458	6.29 ± 0.85 a	2.01 ± 0.34 d	6.02 ± 0.82 a	1.39 ± 0.01 d	4.57 ± 0.26 b	4.06 ± 0.7 bc	3.69 ± 0.08 c	1.86 ± 0.1 d	3.55 ± 0.2 c	4.76 ± 0.1 b	***
**Ethyl-dodecanoate**	1857	0.59 ± 0.09 a	0.46 ± 0.08 b	0.45 ± 0.09 b	0.12 ± 0.001 e	0.25 ± 0.013 cd	0.19 ± 0.04 de	0.47 ± 0.05 b	0.14 ± 0.01 e	0.69 ± 0.1 a	0.36 ± 0.03 bc	***
**Ethyl-tetradecanoate**	2037	0.06 ± 0.002 ab	0.01 ± 0.002 cd	0.04 ± 0.01 b	0.01 ± 0.0001d	0.07 ± 0.02a	0.02 ± 0.002c	0.02 ± 0.001 cd	0.01 ± 0.0005 cd	0.02 ± 0.003 cd	0.02 ± 0.004 cd	***
**Ethyl-hexadecanoate**	2264	0.01 ± 0.004 bc	0.01 ± 0.0009 de	0.01 ± 0.003 b	nd	nd	0.01 ± bc	0.01 ± 0.001 cd	0.01 ± 0.0009 de	0.02 ± 0.003 a	0.010.001 ± b	***
**TERPENES AND NORIISOPRENOIDS**												
**Linalool**	1542	0.003 ± 0.001 b	0.002 ± 0.0006b	0.011 ± 0.002 b	0.003 ± 0.0001 b	0.002 ± 0.0003 b	0.002 ± 0.001b	0.241 ± 0.02 a	0.010 ± 0.0001 b	0.003 ± 0.0007b	0.003 ± 0.001b	***
**β-damascenone**	1755	2.31 ± 0.3e	2.71 ± 0.4 de	3.00 ± 0.5 cde	3.71 ± 0.03 ab	2.71 ± 0.1 de	3.31 ± 0.5 bcd	3.50 ± 0.4 abc	2.91 ± 0.3 cde	4.01 ± 0.5 a	2.61 ± 0.5 de	***
**Citronellol**	1785	0.008	0.008	0.008	0.008	0.009	0.008	0.008	0.009	0.008	0.009	ns
**Nerolidol**	2071	0.006 ± 0.002d	0.008 ± 0.001d	0.009 ± 0.001 cd	0.008 ± 0.0002 cd	0.026 ± 0.007 a	0.015 ± 0.004 b	0.009 ± 0.0009 cd	0.016 ± 0.0005 b	0.010 ± 0.001 cd	0.013 ± 0.003 bc	***
**MISCELLANEOUS**												
**4-vinyl-guaiacol**	2262	0.43 ± 0.06ab	0.20 ± 0.05 f	0.34 ± 0.05 cd	0.18 ± 0.001f	0.23 ± 0.006 ef	0.29 ± 0.04 de	0.25 ± 0.02 ef	0.49 ± 0.02 a	0.40 ± 0.05 bc	0.46 ± 0.08 ab	***
**3- methyl-thio-1-propanol**	1141	3.68 ± 0.56 a	1.04 ± 0.33 ef	2.97 ± 0.37 b	0.69 ± 0.01 fg	1.08 ± 0.14 ef	1.33 ± 0.19 de	2.23 ± 0.01 c	0.31 ± 0.1 g	1.83 ± 0.25 cd	1.29 ± 1.79 e	***

^1^ Different letters in the same row point to significant differences (ANOVA, LSD test). ^2^ IK indicates Kovat’s retention index. ^3^ nd, aroma not detected. ^4^*p*-value < 0.05.

**Table 5:** Analysis of variance of Verdejo and different white crosses during 2018 and 2019 season.

	Acids	Alcohols	Esters	Terpenes	Miscellaneous
Varieties					
Verdejo	19.17 b	263.70 d	8.21 h	2.84 b	3.91 b
MV11	14.91 a	224.60 ab	2.83 a	3.39 d	3.58 ab
MV7	24.91 cd	329.18 f	5.74 e	1.72 a	4.92 d
MV67	21.85 bc	258.01 cd	5.78 d	3.89 e	4.53 cd
MS33	19.54 b	229.75 b	4.78 d	2.96 bc	3.52ab
MS82	25.28 cd	234.77 b	5.20 d	3.88 e	5.08 e
MS30	18.59 ab	216.39 a	3.27 b	3.94 e	3.71ab
MC180	21.78 bc	249.13 c	3.89 c	3.34 cd	4.93 d
MC69	25.91 de	289.51 e	7.62 g	3.41 d	3.16 a
MT103	29.42 e	248.02 c	6.31 f	4.13 e	3. 95 bc
Year					
2018	14.76 a	218.60 a	3.70 a	3.27 a	3.81 a
2019	29.52 b	290.01 b	7.01 b	3.43 a	4.45 b
Interactions					
Variety x Year	**	**	**	**	**

Different letters in the same row point to significant differences (MANOVA, LSD test).

## Supplementary Materials

The following are available online. Table S1: Limit of detection and quantification of the method; Table S2: Sensory aromas with the corresponding attribute studied in the different wines; Table S3: Sensory aromas with the corresponding attribute studied in the different wines in 2018 season; Table S4: Sensory aromas with the corresponding attribute studied in the different wines in 2019 season; Figure S1: Climatic data from years 2018 and 2019 in experimental vineyard during the season of veraison; Figure S2: Chromatogram obtained in the GC-MS analysis for Verdejo wine and MC69 wine.

## References

[1] Salinas M.R., Zalacain A., Pardo F., Alonso G.L. (2004). Stir bar sorptive extraction applied to volatile constituents evolution during Vitis vinifera ripening. J. Agric. Food Chem..

[2] Gonzalez-Viñas M.A., Perez-Coello M.S., Salvador M.D., Cabezudo M.D., Martin-Alvarez P.J. (1996). Changes in gas-chromatographic volatiles of young Airen wines during bottle storage. Food Chem..

[3] Sánchez-Palomo E., Alonso-Villegas R., González-Viñas M.A. (2015). Characterisation of free and glycosidically bound aroma compounds of la Mancha Verdejo white wines. Food Chem..

[4] Ubeda C., Hornedo-Ortega R., Cerezo A.B., Garcia-Parrilla M.C., Troncoso A.M. (2020). Chemical hazards in grapes and wine, climate change and challenges to face. Food Chem..

[5] Intergovernmental Panel on Climate Change (IPCC). https://www.ipcc.ch/sr15/.

[6] Bayo-Canha A., Fernández-Fernández J.I., Martínez Cutillas A., Ruiz-García L. (2014). Genetic analysis of wine grape high-quality ripening in the “Monastrell” × “Syrah” Progeny. Acta Hortic..

[7] Gil-Muñoz R., Moreno-Olivares J.D., Paladines-Quezada D.F., Cebrían-Pérez A., Fernández-Fernández J.I., Ignatio J. (2018). High Anthocyanin Level of Grape Hybrids from Monastrell and Their Wines. Int. J. Hortic. Agric..

[8] Apolinar-Valiente R., Gómez-Plaza E., Terrier N., Doco T., Ros-García J.M. (2017). The composition of cell walls from grape skin in Vitis vinifera intraspecific hybrids. J. Sci. Food Agric..

[9] Gómez-Plaza E., Gil-Muñoz R., Hernández-Jiménez A., López-Roca J.M., Ortega-Regules A., Martínez-Cutillas A. (2008). Studies on the anthocyanin profile of Vitis Vinifera intraspecific hybrids (Monastrell × Cabernet Sauvignon). Eur. Food Res. Technol..

[10] Asproudi A., Ferrandino A., Bonello F., Vaudano E., Pollon M., Petrozziello M. (2018). Key norisoprenoid compounds in wines from early-harvested grapes in view of climate change. Food Chem..

[11] Pons A., Allamy L., Schüttler A., Rauhut D., Thibon C., Darriet P. (2017). What is the expected impact of climate change on wine aroma compounds and their precursors in grape?. OENO One.

[12] Keller M. (2010). Managing grapevines to optimise fruit development in a challenging environment: A climate change primer for viticulturists. Aust. J. Grape Wine Res..

[13] Sadras V.O., Moran M.A., Bonada M. (2013). Effects of elevated temperature in grapevine. I Berry sensory traits. Aust. J. Grape Wine Res..

[14] Robinson A.L., Boss P.K., Solomon P.S., Trengove R.D., Heymann H., Ebeler S.E. (2014). Origins of grape and wine aroma. Part 1. Chemical components and viticultural impacts. Am. J. Enol. Vitic..

[15] Rice S., Tursumbayeva M., Clark M., Greenlee D., Dharmadhikari M., Fennell A.Y., Koziel J. (2019). Effects of Harvest Time on the Aroma of White Wines Made from Cold-Hardy Brianna and Frontenac Gris Grapes Using Headspace Solid-Phase Microextraction and Gas Chromatography-Mass Spectrometry-Olfactometry. Foods.

[16] Jordão A., Vilela A., Cosme F. (2015). From Sugar of Grape to Alcohol of Wine: Sensorial Impact of Alcohol in Wine. Beverages.

[17] Slegers A., Angers P., Pedneault K. (2017). Volatile Compounds from Must and Wines from Five White Grape Varieties. J. Food Chem. Nanotechnol..

[18] Pedneault K., Dorais M., Angers P. (2013). Flavor of cold-hardy grapes: Impact of berry maturity and environmental conditions. J. Agric. Food Chem..

[19] González-Barreiro C., Rial-Otero R., Cancho-Grande B., Simal-Gándara J. (2015). Wine Aroma Compounds in Grapes: A Critical Review. Crit. Rev. Food Sci. Nutr..

[20] Cholet C., Claverol S., Claisse O., Rabot A., Osowsky A., Dumot V., Ferrari G., Gény L. (2016). Tartaric acid pathways in Vitis vinifera L. (cv. Ugni blanc): A comparative study of two vintages with contrasted climatic conditions. BMC Plant Biol..

[21] Tredoux A., de Villiers A., Majek P., Lynen F., Crouch A., Sandra P. (2008). Stir bar sorptive extraction combined with GC-MS analysis and chemometric methods for the classification of south African wines according to the volatile composition. J. Agric. Food Chem..

[22] Sun Q., Sacks G., Lerch S., Heuvel J.E.V. (2011). Impact of shoot thinning and harvest date on yield components, fruit composition, and wine quality of Marechal Foch. Am. J. Enol. Vitic..

[23] Fan W., Shen H., Xu Y. (2011). Quantification of volatile compounds in Chinese soy sauce aroma type liquor by stir bar sorptive extraction and gas chromatography-mass spectrometry. J. Sci. Food Agric..

[24] Sánchez-Palomo E., Delgado J.A., Ferrer M.A., Viñas M.A.G. (2019). The aroma of La Mancha Chelva wines: Chemical and sensory characterization. Food Res. Int..

[25] Etievant P.X., Maarse H. (1991). Volatile Compounds in Foods and Beverages.

[26] SIAM http://siam.imida.es/apex/f?p=101:46:2816839391995198.

[27] Coelho E., Rocha S.M., Barros A.S., Delgadillo I., Coimbra M.A. (2007). Screening of variety- and pre-fermentation-related volatile compounds during ripening of white grapes to define their evolution profile. Anal. Chim. Acta.

[28] Campo E., Cacho J., Ferreira V. (2007). Solid phase extraction, multidimensional gas chromatography mass spectrometry determination of four novel aroma powerful ethyl esters. Assessment of their occurrence and importance in wine and other alcoholic beverages. J. Chromatogr. A.

[29] Esteban-Fernández A., Muñoz-González C., Jiménez-Girón A., Pérez-Jiménez M., Pozo-Bayón M.Á. (2018). Aroma release in the oral cavity after wine intake is influenced by wine matrix composition. Food Chem..

[30] Kalua C.M., Boss P.K. (2010). Comparison of major volatile compounds from Riesling and Cabernet Sauvignon grapes (Vitis vinifera L.) from fruitset to harvest. Aust. J. Grape Wine Res..

[31] Yang Y., Jin G.J., Wang X.J., Kong C.L., Liu J.B., Tao Y.S. (2019). Chemical profiles and aroma contribution of terpene compounds in Meili (Vitis vinifera L.) grape and wine. Food Chem..

[32] Lorenzo C., Pardo F., Zalacain A., Alonso G.L., Salinas M.R. (2008). Differentiation of co-winemaking wines by their aroma composition. Eur. Food Res. Technol..

[33] Kotseridis Y., Baumes R. (2000). Identification of impact odorants in Bordeaux red grape juice, in the commercial yeast used for its fermentation, and in the produced wine. J. Agric. Food Chem..

[34] Wang X., Tao Y.-S., Wu Y., An R., Yue Z.-Y. (2017). Aroma compounds and characteristics of noble-rot wines of Chardonnay grapes artificially botrytized in the vineyard. Food Chem..

[35] Vilanova M., Genisheva Z., Masa A., Oliveira J.M. (2010). Correlation between volatile composition and sensory properties in Spanish Albariño wines. Microchem. J..

[36] Sanchez-Palomo E., Díaz-Maroto M.C., Viñas M.A.G., Soriano-Pérez A., Pérez-Coello M.S. (2007). Aroma profile of wines from Albillo and Muscat grape varieties at different stages of ripening. Food Control.

[37] Styger G., Prior B., Bauer F.F. (2011). Wine flavor and aroma. J. Ind. Microbiol. Biotechnol..

[38] Falcão L.D., de Revel G., Rosier J.P., Bordignon-Luiz M.T. (2008). Aroma impact components of Brazilian Cabernet Sauvignon wines using detection frequency analysis (GC-olfactometry). Food Chem..

[39] Francis I.L., Newton J.L. (2005). Determining wine aroma from compositional data. Aust. J. Grape Wine Res..

[40] Zhang S., Petersen M.A., Liu J., Toldam-Andersen T.B., Ebeler S.E., Hopfer H. (2015). Influence of pre-fermentation treatments on wine volatile and sensory profile of the new disease tolerant cultivar solaris. Molecules.

[41] Ferrandino A., Carlomagno A., Baldassarre S., Schubert A. (2012). Varietal and pre-fermentative volatiles during ripening of Vitis vinifera cv Nebbiolo berries from three growing areas. Food Chem..

[42] Bindon K., Varela C., Kennedy J., Holt H., Herderich M. (2013). Relationships between harvest time and wine composition in Vitis vinifera L. cv. Cabernet Sauvignon 1. Grape and wine chemistry. Food Chem..

[43] Kalua C.M., Boss P.K. (2009). Evolution of volatile compounds during the development of cabernet sauvignon grapes (vitis vinifera l.). J. Agric. Food Chem..

[44] Dennis E.G., Keyzers R.A., Kalua C.M., Maffei S.M., Nicholson E.L., Boss P.K. (2012). Grape contribution to wine aroma: Production of hexyl acetate, octyl acetate, and benzyl acetate during yeast fermentation is dependent upon precursors in the must. J. Agric. Food Chem..

[45] Rapp A., Versini G. (1995). Influence of nitrogen compounds in grapes on aroma compounds of wines. Dev. Food Sci..

[46] Gómez-Míguez M.J., Gómez-Míguez M., Vicario I.M., Heredia F.J. (2007). Assessment of colour and aroma in white wines vinifications: Effects of grape maturity and soil type. J. Food Eng..

[47] Rice S., Lutt N., Koziel J.A., Dharmadhikari M., Fennell A. (2018). Determination of selected aromas in marquette and frontenac wine using headspace-SPME coupled with GC-MS and simultaneous olfactometry. Separations.

[48] Niu Y., Zhu Q., Xiao Z. (2020). Characterization of perceptual interactions among ester aroma compounds found in Chinese Moutai Baijiu by gas chromatography-olfactometry, odor Intensity, olfactory threshold and odor activity value. Food Res. Int..

[49] Gil-Muñoz R., Jiménez-Martínez M.D., Bautista-Ortín A.B., Gómez-Plaza E. (2019). Effect of the use of purified grape pomace as a fining agent on the volatile composition of monastrell wines. Molecules.

[50] Pedroza M.A., Carmona M., Pardo F., Salinas M.R., Zalacain A. (2012). Waste grape skins thermal dehydration: Potential release of colour, phenolic and aroma compounds into wine. CYTA J. Food.

[51] Pardo-García A.I., De La Hoz K.S., Zalacain A., Alonso G.L., Salinas M.R. (2014). Effect of vine foliar treatments on the varietal aroma of Monastrell wines. Food Chem..

[52] Escudero A., Campo E., Fariña L., Cacho J., Ferreira V. (2007). Analytical characterization of the aroma of five premium red wines. Insights into the role of odor families and the concept of fruitiness of wines. J. Agric. Food Chem..

[53] Coelho E., Coimbra M.A., Nogueira J.M.F., Rocha S.M. (2009). Quantification approach for assessment of sparkling wine volatiles from different soils, ripening stages, and varieties by stir bar sorptive extraction with liquid desorption. Anal. Chim. Acta.

[54] Schneider R., Razungles A., Charrier F., Brixs R. (2002). Efect du site, de la maturité et de l’éclairement des grapes sur la composition aromatique des baies de Vitis vinifera L. cv. Melon B. dans le vignoble du Muscadet. Bull. OIV.

[55] Tufariello M., Capone S., Siciliano P. (2012). Volatile components of Negroamaro red wines produced in Apulian Salento area. Food Chem..

[56] Perestrelo R., Fernandes A., Albuquerque F.F., Marques J.C., Câmara J.S. (2006). Analytical characterization of the aroma of Tinta Negra Mole red wine: Identification of the main odorants compounds. Anal. Chim. Acta.

[57] Swiegers J.H., Bartowsky E., Henschke P., Pretorius I.S. (2005). Yeast and bacterial modulation of wine aroma and flavour. Aust. J. Grape Wine Res..

[58] Nicolli K.P., Biasoto A.C.T., Souza-Silva É.A., Guerra C.C., dos Santos H.P., Welke J.E., Zini C.A. (2018). Sensory, olfactometry and comprehensive two-dimensional gas chromatography analyses as appropriate tools to characterize the effects of vine management on wine aroma. Food Chem..

[59] Etschmann M.M.W., Kötter P., Hauf J., Bluemke W., Entian K.D., Schrader J. (2008). Production of the aroma chemicals 3-(methylthio)-1-propanol and 3-(methylthio)-propylacetate with yeasts. Appl. Microbiol. Biotechnol..

[60] Genovese A., Gambuti A., Piombino P., Moio L. (2007). Sensory properties and aroma compounds of sweet Fiano wine. Food Chem..

[61] Chatonnet P., Dubourdieu D., Boidron J.-N., Lavigne V. (1993). Synthesis of volatile phenols by Saccharomyces cerevisiae in wines. J. Sci. Food Agric..

[62] Ugliano M., Moio L. (2008). Free and hydrolytically released volatile compounds of Vitis vinifera L. cv. Fiano grapes as odour-active constituents of Fiano wine. Anal. Chim. Acta.

[63] Qian M.C., Fang Y., Shellie K. (2009). Volatile composition of merlot wine from different vine water status. J. Agric. Food Chem..

[64] Moreno-Olivares J.D., Paladines-Quezada D., Fernández-Fernández J.I., Bleda-Sánchez J.A., Martínez-Moreno A., Gil-Muñoz R. (2020). Study of aromatic profile of different crosses of Monastrell white wines. J. Sci. Food Agric..

[65] Vilanova M., Campo E., Escudero A., Graña M., Masa A., Cacho J. (2012). Volatile composition and sensory properties of Vitis vinifera red cultivars from North West Spain: Correlation between sensory and instrumental analysis. Anal. Chim. Acta.

[66] Ghaste M., Narduzzi L., Carlin S., Vrhovsek U., Shulaev V., Mattivi F. (2015). Chemical composition of volatile aroma metabolites and their glycosylated precursors that can uniquely differentiate individual grape cultivars. Food Chem..

[67] Xu C.H., Chen G.S., Xiong Z.H., Fan Y.X., Wang X.C., Liu Y. (2016). Applications of solid-phase microextraction in food analysis. TrAC Trends Anal. Chem..

[68] Liu J., Zhao W., Li S., Zhang A., Zhang Y., Liu S. (2018). Characterization of the key aroma compounds in proso millet wine using headspace solid-phase microextraction and gas chromatography-mass spectrometry. Molecules.

[69] Gómez-Plaza E., Mestre-Ortuño L., Ruiz-García Y., Fernández-Fernández J.I., López-Roca J.M. (2012). Effect of benzothiadiazole and methyl jasmonate on the volatile compound composition of Vitis vinifera L. Monastrell grapes and wines. Am. J. Enol. Vitic..

[70] Arcari S.G., Caliari V., Sganzerla M., Godoy H.T. (2017). Volatile composition of Merlot red wine and its contribution to the aroma: Optimization and validation of analytical method. Talanta.

