# Supplementary material for: Aromatic Characterization of New White Wine Varieties Made from Monastrell Grapes Grown in South-Eastern Spain
Source: Molecules. 2020 Aug 27;25(17):3917. doi: 10.3390/molecules25173917 (PMC7503703; doi:10.3390/molecules25173917)

molecules

Table A1. Limit of detection and quantification of the method.

Compounds	*RT* (min)	*LD* (mgL^{-1})	*LQ* (mgL^{-1})
Ethyl acetate	*3,00*	*0,0290*	*0,0966*
1-propanol	*6,64*	2,1204	7,0681
2-methyl-1-propanol	*9,10*	0,0615	0,2050
3-methyl-1-butanol-acetate	*9,64*	0,0012	0,0042
3-methyl-1-butanol	*13,90*	0,0175	0,0584
Ethyl hexanoate	*14,72*	0,0162	0,0540
1-hexanol	*20,13*	0,0713	0,2376
Z-3-hexen-1-ol	*21,37*	0,0074	0,0246
Ethyl octanoate	*23,70*	0,0001	0,0002
Linalool	*28,36*	0,00001	0,00002
3-methyl-1-propanol	*34,73*	0,0310	0,1035
β-damascenone	*36,07*	0,1046	0,3488

Citronellol	*36,62*	0,000001	0,000003
Ethyl-dodecanoate	*39,25*	0,0001	0,0003
Hexanoic acid	*39,58*	0,0006	0,0021
β-phenyl-ethanol	*41,71*	0,1236	0,4121
Nerolidol	*45,96*	0,00001	0,00002
Ethyl tetradecanoate	*46,15*	0,0005	0,0018
Octanoic acid	*46,71*	0,0027	0,0091
4-vinyl-guaiacol	*50,74*	0,00002	0,0001
Ethyl hexadecanoate	*52,36*	0,0002	0,0008
Decanoic acid	*53,15*	0,0010	0,0034
9-decenoic acid	*54,91*	0,0032	0,0105

[1] Retention time (min), [2] detection limit (mgL^{-1}), [3] quantification limit (mgL^{-1}).

Table A2. Sensory aromas with the corresponding attribute studied in the different wines.

Attribute	Group	Aroma

Fruity	Citric	lemon/grapefruit/lime/orange
	Seed Fruit	Pear/apple/quince/
	Stone Fruit	Apricot/peach
	Exotic Fruit	pineapple/passion/banana/fruit/mango/lychee/coconut/paraguayan
Floral	White flowers	Almond/white carnation/jasmine/orange blossom
	Colour flowers	Rose/geranium/violet/citronella
Aromatic herbs	Leaves	mint/eucaliptus/peppermint
Ether serie	Yeast	Bread/ stearin
	Other	Acetone/ ripe banana
Chemical serie	Aromas	acetic/alcohol/sulfur/glue/solvent/tar/artichoke
Balsamic serie	Species	Vainilla
	Wood	pine
Spicy serie	Aromas	menthol/ginger/camphor/lavender

able A3. Sensory aromas with the corresponding attribute studied in the different wines in 2018 season. The aroma characteristics have relative standard deviation of below 0.05 (RSD < 0.05).

2018	Verdejo	MT103	MV11	MV67	MV7	MS30	MS33	MS82	MC180	MC69
**Fruity**	1,08 + 0,02e[1]	0,85 + 0,04f	1,46 + 0,07b	1,46 + 0,04b	1,54 + 0,03a	1,31 + 0,03d	1,38 + 0,01c	0,69 + 0,02g	0,64 + 0,05h	1,07 + 0,04e
**Citrics**	1,23 + 0,04a	1,07 + 0,03b	0,77 + 0,02d	0,62 + 0,03e	0,85 + 0,02c	0,54 + 0,05f	0,62 + 0,03e	0,46 + 0,03g	0,36 + 0,04h	0,50 + 0,02f
**Exotic fruit**	1,08 + 0,05b	0,71 + 0,03f	1,08 + 0,04b	1,46 + 0,03a	0,77 + 0,04e	0,69 + 0,04f	0,77 + 0,04d	0,31 + 0,06g	0,79 + 0,01c	0,71 + 0,01f
**Floral**	0,77 + 0,02c	0,29 + 0,02f	0,69 + 0,03d	0,62 + 0,01e	0,77 + 0,01c	0,85 + 0,01b	0,62 + 0,02e	0,77 + 0,04c	0,29 + 0,02f	1,21 + 0,05a
**A. herbs**	0,23 + 0,06f	1,07 + 0,05b	0,69 + 0,04c	0,62 + 0,02d	0,38 + 0,03e	0,62 + 0,03d	0,62 + 0,01d	1,23 + 0,02a	0,71 + 0,03c	1,21 + 0,04a
**Ether**	0,85 + 0,03a	0,07 + 0,01h	0,38 + 0,04f	0,54 + 0,06c	0,62 + 0,02b	0,31 + 0,01g	0,46 + 0,02e	0,46 + 0,01d	0,43 + 0,05e	0,50 + 0,03c
**Chemical**	0,69 + 0,01c	0,93 + 0,06a	0,46 + 0,01d	0,08 + 0,01h	0,38 + 0,01e	0,15 + 0,03g	0,23 + 0,03f	0,92 + 0,02b	0,21 + 0,06f	0,36 + 0,02e
**Balsamic**	0,15 + 0,02e	0,50 + 0,03c	0,23 + 0,03d	0,85 + 0,08a	nd[2]	0,15 + 0,04e	0,15 + 0,04e	0,15 + 0,01e	0,14 + 0,02e	0,57 + 0,03b
**Spice**	0,15 + 0,03e	0,36 + 0,04b	0,15 + 0,05e	0,62 + 0,02a	0,23 + 0,01d	0,31 + 0,06c	0,38 + 0,02b	0,38 + 0,05b	0,21 + 0,03d	0,64 + 0,03a

[1]Different letters in the same row point to significant differences (ANOVA, DUNCAN post-hoc test). [2]nd, aroma not detected.

molecules

Table A4. Sensory aromas with the corresponding attribute studied in the different wines in 2019 season. The aroma characteristics have relative standard deviation of below 0.05 (RSD < 0.05).

2019	**Verdejo**	**MT103**	**MV11**	**MV67**	**MV7**	**MS30**	**MS33**	**MS82**	**MC180**	**MC69**
Fruity	1,75 + 0,02b[1]	2,17 + 0,08a	1,45 + 0,05e	1,50 + 0,06d	1,60 + 0,06c	1,25 + 0,06f	0,83 + 0,04h	1,08 + 0,05g	1,70 + 0,01b	1,50 + 0,05d
Citrics	0,92 + 0,04c	0,75 + 0,03e	0,42 + 0,01h	0,75 + 0,03e	0,67 + 0,04f	1,00 + 0,02b	0,58 + 0,05g	1,17 + 0,05a	0,83 + 0,03d	0,67 + 0,03f
Exotic fruit	0,67 + 0,05e	0,75 + 0,04c	0,50 + 0,02f	0,67 + 0,04d	0,75 + 0,05c	0,33 + 0,01h	0,42 + 0,02g	0,42 + 0,04g	1,00 + 0,04a	0,83 + 0,03b
Floral	0,97 + 0,05d	1,40 + 0,06a	0,58 + 0,06i	0,92 + 0,05e	0,67 + 0,06h	0,83 + 0,03f	0,75 + 0,04g	1,00 + 0,06c	0,75 + 0,06g	1,17 + 0,04b
A. herbs	0,42 + 0,03d	0,50 + 0,02c	0,50 + 0,04c	0,08 + 0,03f	0,33 + 0,02e	0,67 + 0,04b	0,33 + 0,02e	0,42 + 0,01d	0,50 + 0,04c	1,42 + 0,05a
Ether	0,25 + 0,02c	0,08 + 0,01f	0,58 + 0,03a	0,42 + 0,01b	0,42 + 0,02b	0,08 + 0,03f	0,17 + 0,03d	0,17 + 0,03e	0,08 + 0,01f	0,17 + 0,03e
Chemical	0,25 + 0,04b	0,58 + 0,02a	nd[2]	nd	nd	nd	0,08 + 0,01c	0,08 + 0,01c	0,25 + 0,03b	0,08 + 0,04c
Balsamic	0,17 + 0,03e	0,46 + 0,03c	0,17 + 0,01e	0,17 + 0,01e	0,08 + 0,01f	0,50 + 0,02b	0,08 + 0,02f	0,73 + 0,03a	0,08 + 0,02f	0,25 + 0,03d
Spice	0,08 + 0,01g	0,62 + 0,04c	0,08 + 0,02g	0,25 + 0,05e	nd	0,58 + 0,01d	0,92 + 0,02b	1,08 + 0,05a	0,08 + 0,03g	0,17 + 0,05f

[1]Different letters in the same row point to significant differences (ANOVA, DUNCAN post-hoc test). [2]nd, aroma not detected.

Figure A1. Climatic data from years 2018 and 2019 in experimental vineyard during the season of veraison.

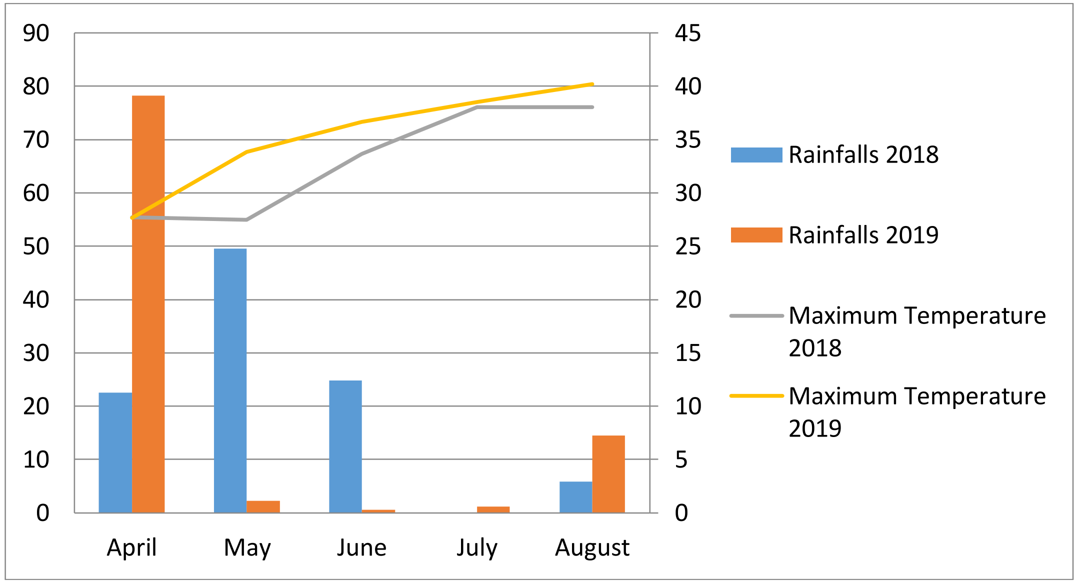

Figure A2. Chromatogram obtained in the GC-MS analysis for Verdejo wine and MC69 wine.

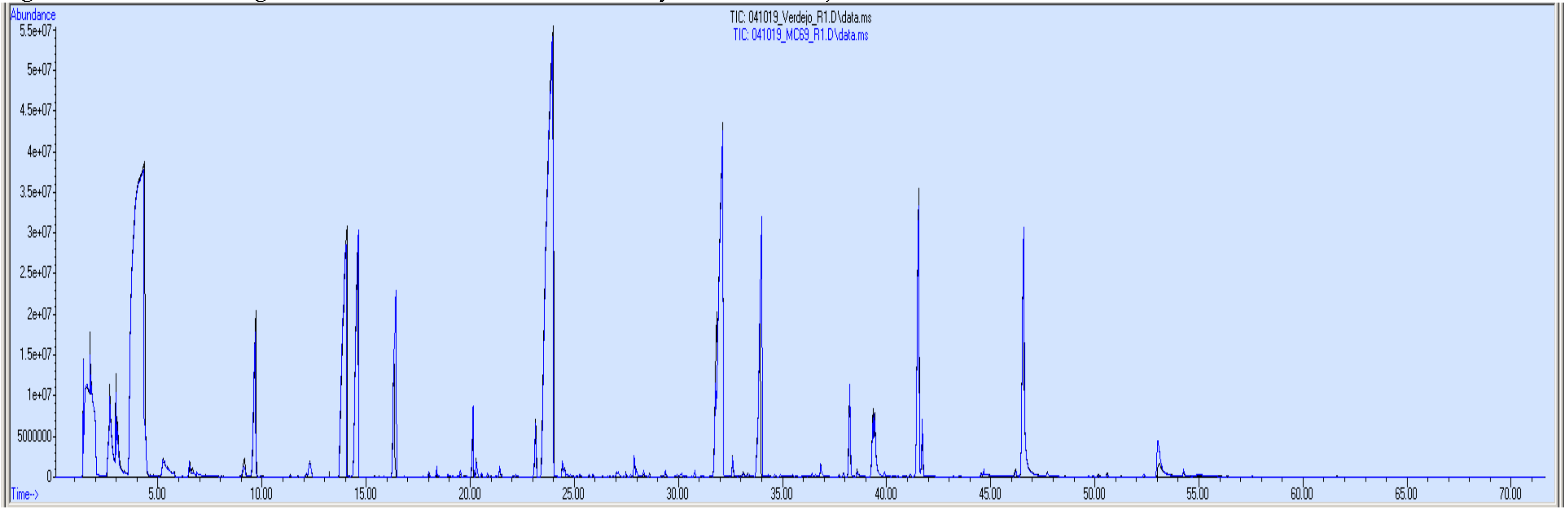

Supplement: Supplementary file 1 [file molecules-25-03917-s001.pdf]
